# Exploring brain perfusion in dogs with meningoencephalitis of unknown origin: A promising role for arterial spin labeling imaging

**DOI:** 10.1111/jvim.17259

**Published:** 2024-12-10

**Authors:** Léa Arti, Kirsten Gnirs, Stella Papageorgiou, Yannick Ruel, Albert Agoulon, Nathalie Boddaert, Hugues Gaillot

**Affiliations:** ^1^ Unit of Neurology Centre Hospitalier Vétérinaire ADVETIA Vélizy‐Villacoublay France; ^2^ Unit of Diagnostic Imaging Centre Hospitalier Vétérinaire ADVETIA Vélizy‐Villacoublay France; ^3^ INRAE, Oniris, UMR BIOEPAR Nantes F‐44300 France; ^4^ Paediatric Radiology Department AP‐HP, Hôpital Necker Enfants Malades, Université de Paris Paris F‐75105 France; ^5^ Institut Imagine INSERM U1163, Universié de Paris Paris F‐75015 France

**Keywords:** ASL, canine, magnetic resonance imaging, MRI, MUO, PWI

## Abstract

**Background:**

Arterial spin labeling (ASL) is a noninvasive brain perfusion magnetic resonance imaging (MRI) technique that has not been assessed in dogs with meningoencephalitis of unknown origin (MUO).

**Hypothesis/Objectives:**

Assess brain perfusion changes characteristics before and after medical treatment, and investigate the role of ASL perfusion in the diagnosis and prognosis of MUO in dogs.

**Animals:**

Thirty‐one dogs with presumed MUO.

**Methods:**

Prospective study. Each animal had brain MRI including standard and ASL perfusion sequences at presentation and after treatment of 3 months or longer. Brain perfusion characteristics were assessed visually and by cerebral blood flow (CBF) measurements. Perfusion characteristics were compared pre‐ and post‐treatment.

**Results:**

Dogs with MUO had preferential localization of lesions in optic nerves (ONs) and brainstem. At presentation, one third of the dogs with MUO had focal brain perfusion alterations and two‐thirds had global brain hypoperfusion. Both focal and global brain perfusion changes resolved after treatment in all surviving dogs. Arterial spin labeling failed to predict prognosis.

**Conclusions and Clinical Importance:**

Brain ASL perfusion in dogs with MUO demonstrated the value of ASL in the diagnosis and follow‐up of the condition, suggesting the value of adding ASL to the clinical evaluation in dogs with suspected MUO. Preferential lesion localization in ON and brainstem resembled findings in the central nervous system of human patients with inflammatory demyelinating diseases. Future studies with histopathological confirmation are needed to better characterize the benefits of ASL in the different subtypes of non‐infectious encephalomyelitis in dogs.

**Abbreviations**
3D‐pCASL: three‐dimensional pseudocontinuous ASL
ADC: apparent diffusion coefficient
ASL: arterial spin labeling
ASL‐ctrl: control ASL
ASL‐T0: initial ASL
CBF: cerebral blood flow
CNS: central nervous system
CSF: cerebrospinal fluid
DWI: diffusion‐weighted imaging
FLAIR: fluid‐attenuated inversion recovery
FSE: fast spin echo
GME: granulomatous meningoencephalitis
ICP: intracranial pressure
IIP: increased intracranial pressure
IQR: interquartile range
MRI: magnetic resonance imaging
MRI‐ctrl: control MRI
MRI‐T0: initial MRI
MUO: meningoencephalitis of unknown origin
NE: necrotizing encephalitis
NIEM: non‐infectious encephalomyelitis
NLE: necrotizing leucoencephalitis
NME: necrotizing meningoencephalitis
ON: optic nerve
PACS: picture archiving communication system
PLD: post‐labeling delay
rCBF: relative CBF
ROI: region of interest
SC: spinal cord
SWI: susceptibility‐weighted imaging
T1W: T1‐weighted
T2W: T2‐weighted
TNCC: total nucleated cell count

## 1 INTRODUCTION

Meningoencephalitis of unknown origin (MUO) is nomenclature used in dogs to describe non‐infectious, most likely immune‐mediated, inflammatory diseases of the central nervous system (CNS) including granulomatous meningoencephalitis (GME), necrotizing meningoencephalitis (NME), and necrotizing leukoencephalitis (NLE). These meningoencephalitides are suspected based on clinical findings but can neither be confirmed nor distinguished from one another without histopathological analysis, making the term MUO clinically relevant.^1^, ^2^, ^3^ To date, the presumptive antemortem diagnosis of MUO is based on signalment, clinical signs of a CNS disorder, abnormalities on conventional magnetic resonance imaging (MRI), inflammatory cerebrospinal fluid (CSF) findings and negative test results for infectious diseases.^4^, ^5^ Previous studies have described in detail the features of MUO in dogs on conventional MRI and evaluated the prognostic value of MRI.^5^, ^6^, ^7^, ^8^, ^9^, ^10^, ^11^, ^12^, ^13^, ^14^, ^15^, ^16^, ^17^, ^18^, ^19^

Arterial spin labeling (ASL) is a powerful, noninvasive MRI perfusion technique for quantitative evaluation of cerebral blood flow (CBF). It uses magnetically labeled arterial blood protons as a diffusible endogenous tracer and therefore does not require any administration of contrast agent.^20^, ^21^, ^22^, ^23^

In neuroradiology in human medicine, ASL is currently widely applied in the diagnostic evaluation of many brain diseases, in particular infectious and inflammatory encephalitides.^24^, ^25^, ^26^, ^27^, ^28^, ^29^, ^30^, ^31^, ^32^ In viral encephalitis of humans, ASL has identified perfusion changes correlated with the stage of disease, clinical status including seizures, and clinical outcome, and has been proposed as a helpful tool for noninvasive brain function monitoring.^33^, ^34^, ^35^, ^36^, ^37^ In autoimmune encephalitis of humans, recent clinical studies have identified ASL as a suitable early imaging biomarker preceding laboratory diagnostic findings and conventional MRI abnormalities, and as a mean of assessing therapeutic response and pathogenesis of the condition.^38^, ^39^, ^40^, ^41^, ^42^, ^43^, ^44^ In immune‐mediated inflammatory demyelinating CNS diseases of humans, brain ASL perfusion has been much less investigated and its usefulness remains to be determined.^45^, ^46^, ^47^, ^48^, ^49^

Brain ASL‐MRI perfusion is not commonly used in clinical neuroimaging of dogs and very little information can be found in the literature. Arterial spin labeling has been implemented in a dog with suspected late subacute cortical laminar necrosis using a 3 Tesla MRI scanner.^50^ More recently, a prospective study performed on 314 client‐owned dogs and cats has demonstrated that a 3‐dimensional pseudocontinuous ASL (3D‐pCASL) sequence using a 1.5 Tesla MRI scanner can be implemented successfully in dogs.^51^

Considering uncertainty about the pathophysiology of MUO in dogs and the available data on brain perfusion MRI in humans with encephalitis, we hypothesized that 3D‐pCASL would provide useful information on the diagnosis and follow‐up of MUO in dogs. The aims of our study were to (a) assess changes in brain perfusion in dogs with MUO by applying 3D‐pCASL, (b) assess changes in perfusion characteristics after treatment, and (c) investigate the role of ASL perfusion in the diagnosis and prognosis of MUO in dogs.

## 2 MATERIALS AND METHODS

### 2.1 Study design and timing

Ours was a single center (Centre Hospitalier Vétérinaire ADVETIA), prospective, analytical observational study, approved by the ethics committee Jacques Bonnod of VetAgro Sup (registration number 2109) and performed with informed owner consent.

All dogs were recruited between September 2020 and July 2023, from neurology consultations at ADVETIA hospital and were examined by either a board‐certified veterinary neurologist (K.G. or S.P., Diplomates of the European College of Veterinary Neurology [ECVN]) or a third‐year ECVN resident (L.A.). Dogs with a presumed diagnosis of MUO based on previously published guidelines that presented with the inclusion criteria listed in Table 1 were selected.^4^ Final decisions for subject inclusion were made by the neurologists and a board‐certified veterinary radiologist (H.G., Diplomate of the European College of Veterinary Diagnostic Imaging [ECVDI]).

**TABLE 1:** Inclusion criteria of the study.

1.	Age	Dogs older than 6 months
2.	Neurological signs	Compatible with meningoencephalitis
3.	CSF MRI‐T0	TNCC >5 WBC/μL and >50% mononucleated cells and/or Abnormalities suggestive of meningoencephalitis
4.	CSF infectious tests	Negative result for *Neospora caninum* Negative result for distemper virus if dog is not vaccinated
5.	Treatment	Initiated after MRI‐T0 with prednisolone at immunosuppressive dose, and cytarabine or cyclosporin
6.	Re‐examination	During at least 3 months after MRI‐T0 Clinical response assessment CSF recheck left at the clinician discretion Medical treatment adjustment
7.	MRI‐ctrl	After at least 3 months of treatment
8.	MRI protocols	Including conventional MRI and ASL perfusion sequences

Abbreviations: ASL, arterial spin labeling; CSF, cerebrospinal fluid; MRI, magnetic resonance imaging; MRI‐ctrl, control MRI; MRI‐T0, initial MRI; TNCC, total nucleated cell count; WBC, white blood cell.

### 2.2 Magnetic resonance imaging

Magnetic resonance images were obtained using a 1.5 T MR unit (Signa Explorer SV25; GE Medical Systems, Milwaukee, Wis) with a 16‐channel flex coil. The brain MRI protocol included the following conventional pulse sequences: 3D T1‐weighted (T1W), 2D or 3D T2‐weighted (T2W), 3D T2W fluid‐attenuated inversion recovery (FLAIR), 3D susceptibility‐weighted imaging (SWI), diffusion‐weighted imaging (DWI) with apparent diffusion coefficient (ADC) map, and gadolinium‐enhanced 3D T1W (Supporting information S1). Additionally, each MRI protocol included a 3D‐pCASL perfusion imaging pulse sequence that was acquired in a transverse plane before IV injection of gadolinium (Supporting information S2). The post‐labeling delay (PLD) was adjusted according to the dog's weight as previously recommended.^51^ Cerebral blood flow color‐coded maps were generated and registered to anatomical T1W images. All images were stored in a picture archiving communication system (PACS) and analyzed by using a dedicated medical image viewer (Vue PACS, version 12.1.6; Philips, Amsterdam, Netherlands).

### 2.3 Data collection

The following data were extracted from the dogs' medical records by L.A.: breed, sex, age, weight, owner complaint, duration of clinical signs before investigation, physical and neurological examination findings, presence of seizures, time between last seizure and initial MRI examination (MRI‐T0), CSF analysis results, serum biochemistry profile and CBC results when available, treatment, neurologic deficits monitoring relative to the previous visit, follow‐up CSF total nucleated cell count (TNCC), time between MRI‐T0 and control MRI (MRI‐ctrl) obtained after at least 3 months of medical treatment, spontaneous death or euthanasia, duration of follow‐up until death or at time of data acquisition.

Images were analyzed by H.G. and a neuroradiologist with 25 years of experience in human pediatric neuro‐imaging (N.B.). Final decisions on the imaging characteristics were reached collegially on a consensus basis.

Conventional brain MRIs were classified as either normal or pathological. Signal abnormalities were described according to their localization, size, intensity relative to adjacent parenchyma, and morphology as follows: punctiform, nodular (round‐shaped, diameter <5 mm), lenticular (lens‐shaped), mass (tumor‐like), large areas (blurred contour, diameter >1 cm), and diffusely hemispherical. The presence of contrast enhancement after gadolinium injection was recorded.

The quality of ASL images was assessed by visual inspection according to previous recommendations.^51^ Any obvious asymmetry of signal intensity was confirmed by measuring CBF bilaterally and a difference in CBF between both sides <15% was considered not clinically relevant. For each CBF measurement, 3 round 2‐dimensional regions of interest (ROIs; 6‐23 mm^2^) were manually drawn and a mean value was recorded. The presence of a cervical microchip‐related labeling artifact was recorded according to previous characterization.^51^ The approximate distance between the foramen magnum and the artifact was recorded.

The ASL perfusion pattern of each brain lesion was first described visually as hypointense, isointense or hyperintense relative to perilesional or contralateral normal‐appearing parenchyma. Brain regions with hyperintense or hypointense signal were regarded as focal hyperperfusion or focal hypoperfusion, respectively. Additionally, a quantitative evaluation of perfusion was performed by measuring CBF. To minimize interindividual differences in baseline CBF, we calculated relative CBF (rCBF) defined as the mean CBF of a lesion divided by the mean CBF of the normal‐appearing contralateral parenchyma. Suspected focal advanced necrosis with cavitation (T1W hypointense with no enhancement, T2W hyperintense with partial or complete suppression on FLAIR) or focal atrophy (T2W and FLAIR hyperintense with adjacent ventricle enlargement, pericerebral subarachnoid space widening or midline shift toward the lesion) were excluded from ASL perfusion analysis.

Optic nerve (ON) and cervical spinal cord (SC) lesions as indicated by any area of FLAIR hyperintensity or contrast enhancement were recorded.

Follow‐up MRIs were analyzed using the same definitions and methods as described above. Lesion evolution was scored as follows: worsening (enlargement of lesion or appearance of new lesions), unchanged state, partial regression (decrease in lesion number or size or both) or complete disappearance.

Global brain perfusion was assessed before and after treatment by measuring absolute CBF in normal‐appearing areas (cerebral cortex, caudate nuclei, thalamic nuclei, mesencephalic nuclei, and cerebellar cortex).

### 2.4 Statistical analysis

Data analysis was conducted by a veterinary faculty with advanced training in statistics (A.A.), using R version 4.3.3 (https://www.R-project.org/). Data were assessed for normality using the Shapiro‐Wilk test. Continuous data were expressed as medians, interquartile ranges (IQR) and ranges, and compared using the Wilcoxon signed‐rank test for repeated measurements, and the Wilcoxon rank‐sum test for independent measurements. Categorical data were expressed as ratios and percentages, and compared using Fisher's exact test for proportions. Differences were considered significant for *P* < .05.

## 3 RESULTS

### 3.1 Study population

Thirty‐one dogs were included. Breeds represented were mostly small (29/31 dogs weighing <15 kg) and included Yorkshire Terrier (8/31), Chihuahua (5/31), French Bulldog (4/31), Shih Tzu (3/31), and American Staffordshire Terrier, Bichon Maltais, Cairn Terrier, Little Brabançon, Pinscher, Pug, Pomeranian Spitz, Tibetan Spaniel, West Highland White Terrier, Papillon, mixed‐breed (1 each). Seventeen of 31 (55%) dogs were males (3 neutered) and 14/31 (45%) were females (13 spayed). Median age was 5 years (IQR, 4.0‐7.0; range, 0.9‐13.0) and median body weight was 4.4 kg (IQR, 4.0‐8.0; range, 2.6‐36.0).

**FIGURE 1:**
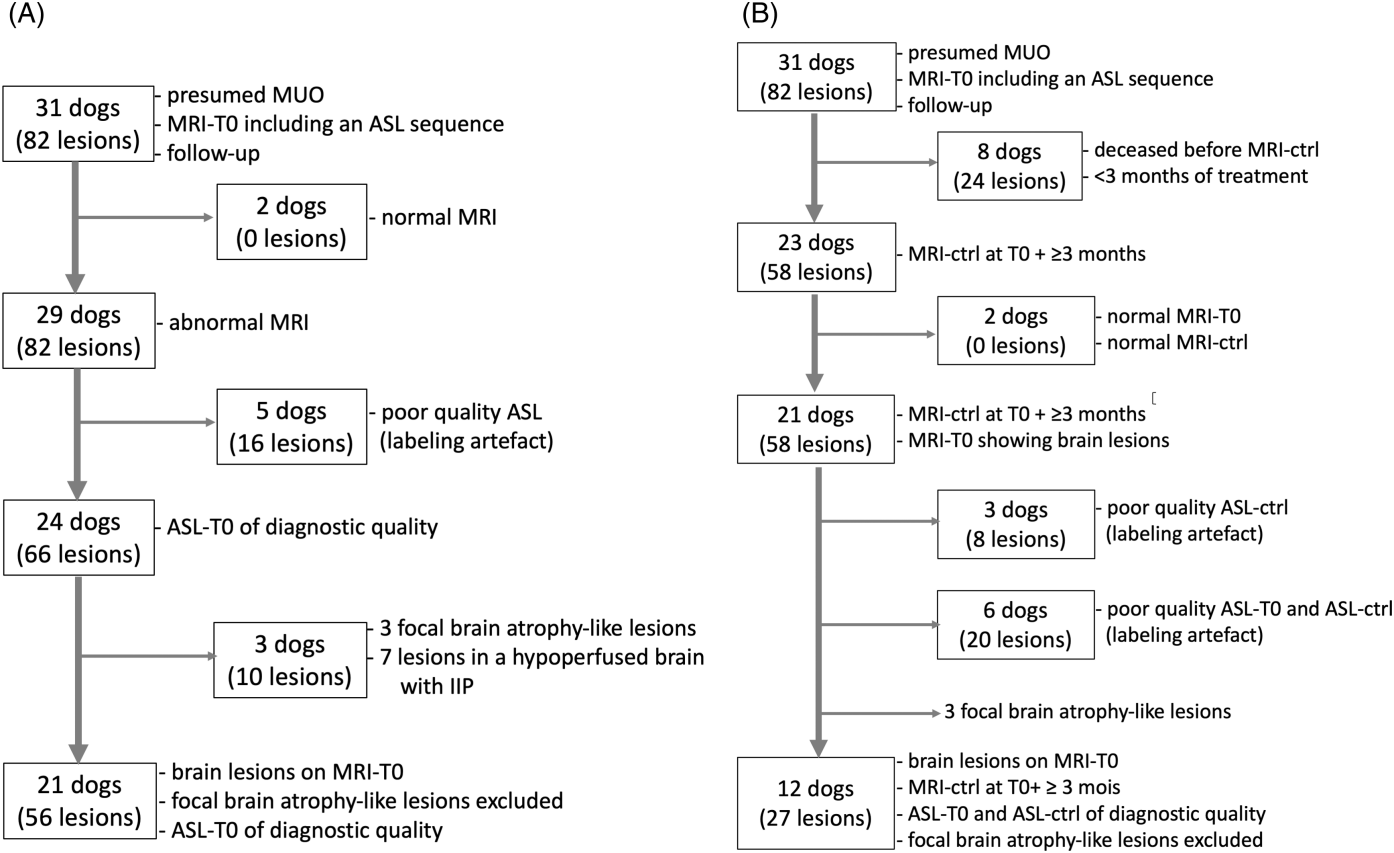
(A) Brain MRI at disease onset (MRI‐T0). (B) Follow‐up brain MRI (MRI‐ctrl). ASL, arterial spin labeling; IIP, increased intracranial pressure; MRI, magnetic resonance imaging; MRI‐ctrl, control MRI; MRI‐T0, initial MRI; MUO, meningoencephalitis of unknown origin.

Two flowcharts of the study, 1 for each MRI examination, are provided (Figure 1).

### 3.2 Clinical manifestations and CSF examination in 31 dogs with MUO

Median duration of clinical signs before presentation was 7 days (range, 12 hours‐4 months; Supporting information S3). Most common neurological signs included cranial nerve deficits (16/31, 52%), ataxia (14/31, 45%), pain (9/31, 29%), reluctance to walk (7/31, 23%), head tilt or head turn (7/31, 23%), and seizures (6/31, 19%). Clinical signs were mainly suggestive of multifocal (19/31, 61%) lesions, located in the forebrain (13/31, 43%) or cerebellum (10/31, 33%). Total nucleated cell count of CSF was increased (>5 WBC/μL) in 28/31 (90%) dogs.

### 3.3 Initial conventional MRI in 29 dogs with MUO and brain lesions

**FIGURE 2:**
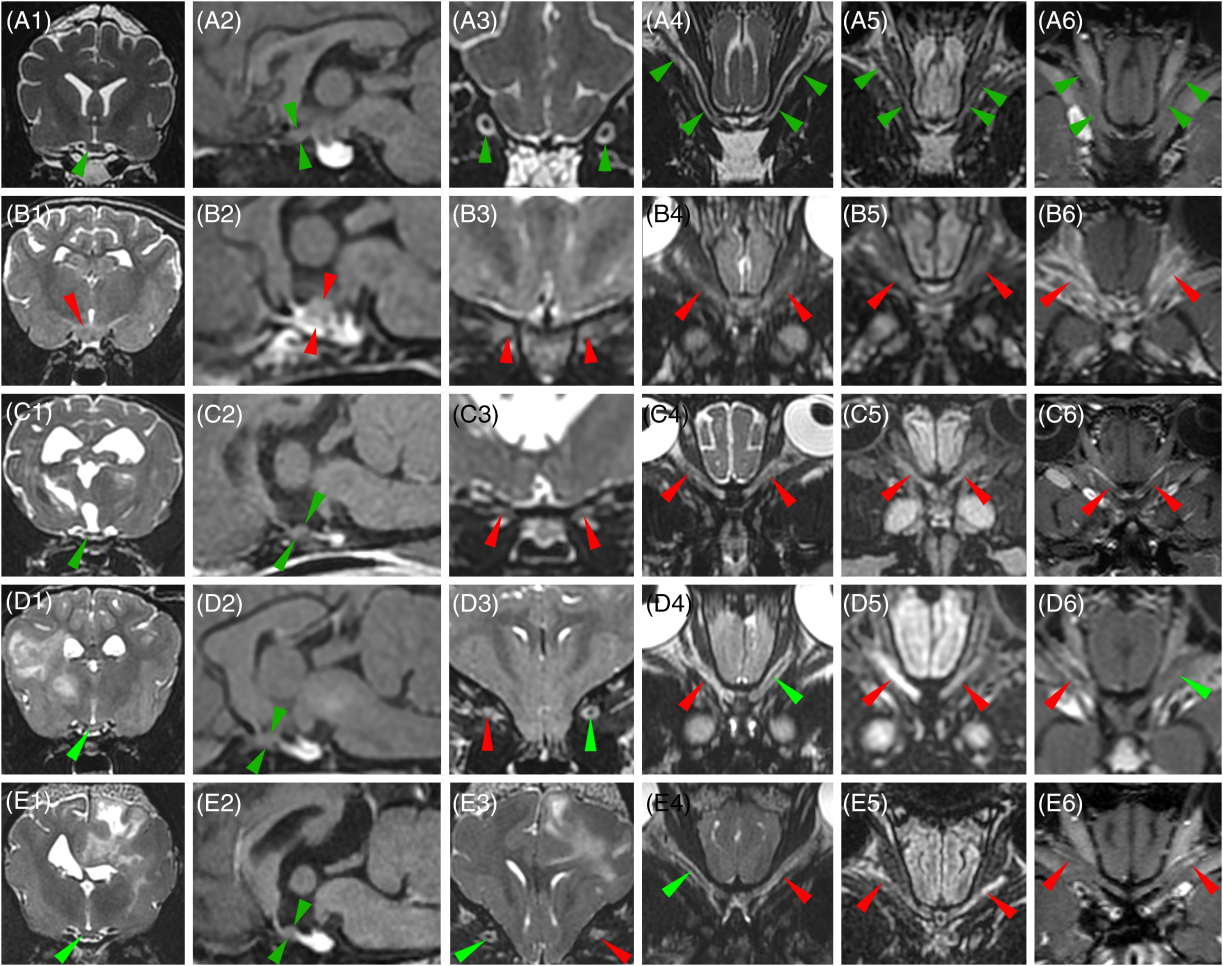
MRI of the optic nerves and chiasm in 4 dogs with a diagnosis of MUO, a 5‐year‐old female Shih Tzu (B1‐B6), 3‐year‐old male Chihuahua (C1‐C6), 4‐year‐old female Shih Tzu (D1‐D6) and 4‐year‐old male French Bulldog (E1‐E6), and in a 7‐year‐old Siberian Husky with normal MRI (A1‐A6) presented as a reference, including transverse T2W images at the level of the optic chiasm (column 1), sagittal fat‐saturated contrast T1W images at the level of the optic chiasm (column 2), transverse T2W images at the level of the retrobulbar segment of the optic nerves (column 3), dorsal oblique images of the optic nerves on T2W (column 4), FLAIR (column 5), and fat‐saturated contrast T1W (column 6) images. Optic structures with normal signal intensity are pointed by green arrow heads and optic structures with abnormal signal intensity are pointed by red arrow heads. One dog with MUO has a T2W hyperintense moderately enhancing 6‐mm‐high mass at the optic chiasm (B1, B2, red arrow heads). The 3 remaining dogs with MUO show normal‐appearing optic chiasm that is under 2.5 mm in height (C1, C2, D1, D2, E1, E2, green arrow heads). All optic nerves in the 4 dogs with MUO display a FLAIR hyperintensity, either generalized (B5, C5, D5, red arrow heads) or segmental (E5), and either symmetrical (B5, C5) or asymmetrical (D5, E5). Most of the optic nerves show variable degrees of T2W hyperintensity (B3, B4, C3, C4, D3, D4, E3, E4, red arrow heads) leading to an attenuation (C4, D4, E4) or complete loss (B4) of the demarcation between the nerve and the peripheral T2W hyperintense CSF. Most of the 4 dogs with MUO display different patterns of contrast enhancement including: Marked and heterogeneous enhancement of the nerves and adjacent structures (B6, red arrow heads), diffuse or segmental enhancement of the outline of the nerves (C6, D6, E6). Only 1 optic nerve does not show any contrast enhancement (D6, left green arrow head); it was moderately FLAIR hyperintense (D5, left red arrow head). Note that all images obtained in a transverse or dorsal plane are displayed with the right side of the dog on the left side of the image and the left side of the dog on the right side of the image. CSF, cerebrospinal fluid; FLAIR, fluid‐attenuated inversion recovery; MRI, magnetic resonance imaging; MUO, meningoencephalitis of unknown origin; T1W, T1‐weighted; T2W, T2‐weighted.

Two of 31 (6%) dogs with MUO had no lesions on conventional MRI. The remaining 29 dogs had a total of 82 lesions (Figure 1). The most common localizations of brain lesions were the cerebral hemispheres (32/82 [39%] lesions, 17/29 [59%] dogs) and the brainstem (25/82 [30%] lesions, 16/29 [55%] dogs; Table 2). No significant association was found between cortical and subcortical localization of lesions (cortical lesions with suspected extension into the adjacent peripheral white matter) and the occurrence of seizures (Fisher's exact test, *P* = .06). Lesions of the ON (Figure 2) were observed in 12/31 (39%) dogs. All of these dogs had clinical visual dysfunction at initial presentation. Twenty one of 31 (68%) dogs had brainstem or ON lesions or both. Lesions in the region of the cervical SC that were included in the imaged area were identified in 9/31 (29%) dogs either on FLAIR or T1 post‐contrast.

**TABLE 2:** Imaging features on initial conventional MRI in dogs with MUO and intracranial lesions or optic nerve lesions.

Intracranial lesions distribution	Lesions, n = 82	Dogs, n = 29
Supratentorial location	48/82 (59%)	
Cortical‐subcortical parenchyma	20/82 (24%)	13/29 (45%)
Deep white matter	12/82 (15%)	9/29 (31%)
Hippocampus	3/82 (4%)	3/29 (10%)
Thalamus	11/82 (13%)	10/29 (34%)
Optic chiasm	2/82 (2%)	2/29 (7%)
Infratentorial location	34/82 (41%)	
Cerebellum	9/82 (11%)	7/29 (24%)
Brainstem	25/82 (30%)	16/29 (55%)
Mesencephalon	12/82 (15%)	10/29 (34%)
Pons	11/82 (13%)	12/29 (41%)
Medulla oblongata	2/82 (2%)	2/29 (7%)
Number and symmetry of lesions		
Single lesion	‐	6/29 (21%)
Multiple asymmetrical lesions	‐	21/29 (72%)
Multiple symmetrical lesions	‐	2/29 (7%)

Intracranial lesions morphology	Lesions, n = 82	Dogs, n = 29
Large area	60/82 (73%)	26/29 (90%)
Mass	10/82 (12%)	9/29 (31%
Nodular	7/82 (9%)	5/29 (17%)
Lenticular	2/82 (2%)	2/29 (7%)
Punctiform	1/82 (1%)	1/29 (3%)
Diffuse brain hemisphere	2/82 (2%)	1/29 (3%)

ON lesions	ON, n = 62	Dogs, n = 31
Retrobulbar segment of ON	21/62	12/31 (39%)
Bilateral ON lesions	18/21	9/12
Unilateral ON lesions	3/21	3/12

Abbreviations: MRI, magnetic resonance imaging; MUO, meningoencephalitis of unknown origin; ON, optic nerve.

The 82 brain lesions were predominantly T2W hyperintense (80/82, 98%), FLAIR hyperintense (79/82, 97%), T1W hypointense (70/82, 85%), DWI hyperintense or isointense (38/80, 48% for each pattern), hyperintense on ADC map (63/80, 79%). None of the lesions were signal void on SWI (Table 3). Contrast enhancement was present in 38/82 (46%) lesions with variable extension, and meningeal enhancement was noted in 2/29 (7%) dogs.

**TABLE 3:** Imaging features on initial conventional MRI in 29 dogs with MUO and brain lesions: Signal intensity and enhancement.

	Lesions *n* = 82	Dogs *n* = 29
Brain lesions signal intensity		
T2W hyperintense	80/82 (98%)	29/29 (100%)
T2W isointense	1/82 (1%)	1/29 (3%)
T2W hypointense	1/82 (1%)	1/29 (3%)
FLAIR hyperintense	79/82 (96%)	29/29 100%
FLAIR isointense	2/82 (2%)	2/29 (7%)
FLAIR hypointense	1/82 (1%)	1/29 (3%)
T1W hyperintense	0/82 (0%)	0/29 (0%)
T1W isointense	12/82 (15%)	9/29 (31%)
T1W hypointense	70/82 (85%)	26/29 (90%)
SWI hypointense	0/82 (0%)	0/29 (0%)
DWI hyperintense	38/80 (48%)	17/28 (61%)
DWI isointense	38/80 (48%)	25/28 (89%)
DWI hypointense	4/80 (5%)	3/28 (11%)
DWI missing data	2/82	1/29
ADC values increased	63/80 (79%)	28/28 (100%)
ADC values not modified	8/80 (10%)	5/28 (18%)
ADC values decreased	9/80 (11%)	5/28 (18%)
ADC values missing data	2/82	1/29
Enhancement pattern		
No lesions enhancement	44/82 (54%)	8/29 (28%)
Enhancement <25% of lesion volume	12/82 (15%)	9/29 (31%)
Enhancement of 25%‐75% of lesion volume	22/82 (27%)	13/29 (45%)
Enhancement >75% of lesion volume	4/82 (5%)	4/29 (14%)
Meningeal enhancement	–	2/29 (7%)

Abbreviations: ADC, apparent diffusion coefficient; DWI, diffusion‐weighted imaging; FLAIR, fluid‐attenuated inversion recovery; MRI, magnetic resonance imaging; MUO, meningoencephalitis of unknown origin; SWI, susceptibility‐weighted imaging; T1W, T1‐weighted; T2W, T2‐weighted.

### 3.4 Initial conventional MRI and ASL perfusion in 21 dogs with MUO and brain lesions

**FIGURE 3:**
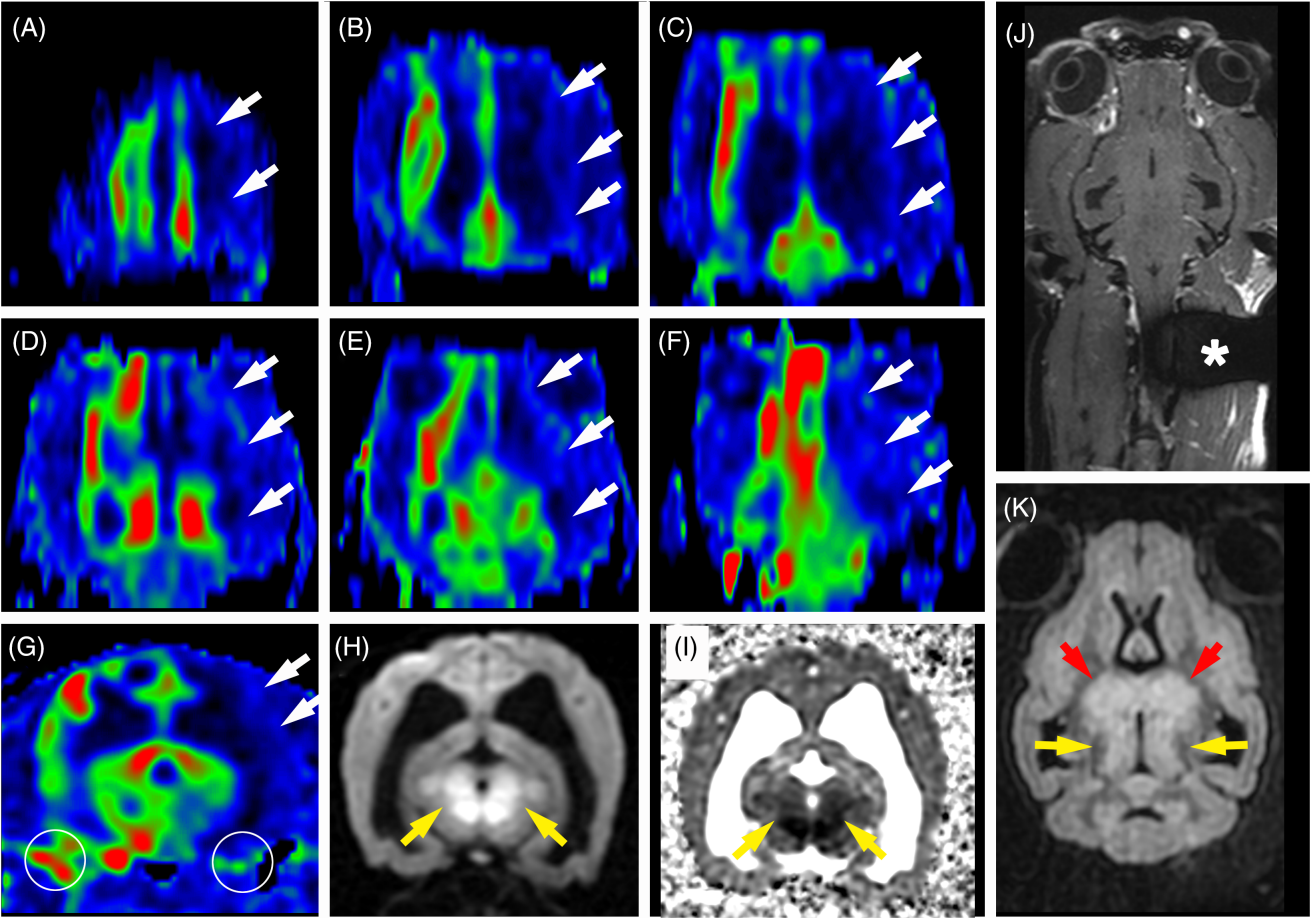
Dorsal reconstructed ASL CBF color maps of the entire brain (A‐F), transverse ASL CBF color map (G), DWI b‐1000 (H) and ADC map (I) at the level of the mesencephalon, and dorsal fat‐saturated contrast (gadolinium‐DTPA) T1W (J) and FLAIR (K) images at the level of the ventral mesencephalon, in a 2‐year‐old Chihuahua weighting 3.5 kg with a diagnosis of MUO. (A‐G) Absence of ASL perfusion signal within the left telencephalon (white arrows) that corresponds to the arterial territory of the left internal carotid artery in dogs. (G) Note the low ASL perfusion signal in branches of the left external carotid artery compared with contralateral arteries (circles). DWI (H) and ADC map (I) are normal in the telencephalic territory of the left internal carotid artery supporting interpretation of the lack of ASL signal within the left telencephalon as an artifact. Note the bilateral symmetrical diencephalic (red arrows) and mesencephalic (yellow arrows) lesions that are hyperintense on FLAIR (K), display a restricted diffusion pattern on DWI and ADC map (H, I) and did not show contrast enhancement (J), that were interpreted as inflammatory brain lesions. (J) Large susceptibility artifact on the dorsal fat‐saturated contrast T1W image (asterisk) most likely because of an identification microchip responsible for a poor labeling because of susceptibility effects. Note that all images obtained in a transverse or dorsal plane are displayed with the right side of the dog on the left side of the image and the left side of the dog on the right side of the image. ADC, apparent diffusion coefficient; ASL, arterial spin labeling; CBF, cerebral blood flow; DWI, diffusion‐weighted image; FLAIR, fluid‐attenuated inversion recovery; MUO, meningoencephalitis of unknown origin; T1W, T1‐weighted.

Eight of 29 dogs were excluded from the ASL perfusion study of MUO brain lesions because of a lack of perfusion signal on visual assessment of CBF color‐coded maps (Figure 1). Five of 8 dogs showed a lack of perfusion signal limited to the left prosencephalon that was interpreted as a labeling failure artifact because of a cervical microchip (Figure 3). These dogs weighed <6 kg, showed a susceptibility artifact in the left‐ventral cervical tissues <5 cm away from the foramen magnum, and had low‐to‐no ASL signal in the ipsilateral external carotid artery branches.

**FIGURE 4:**
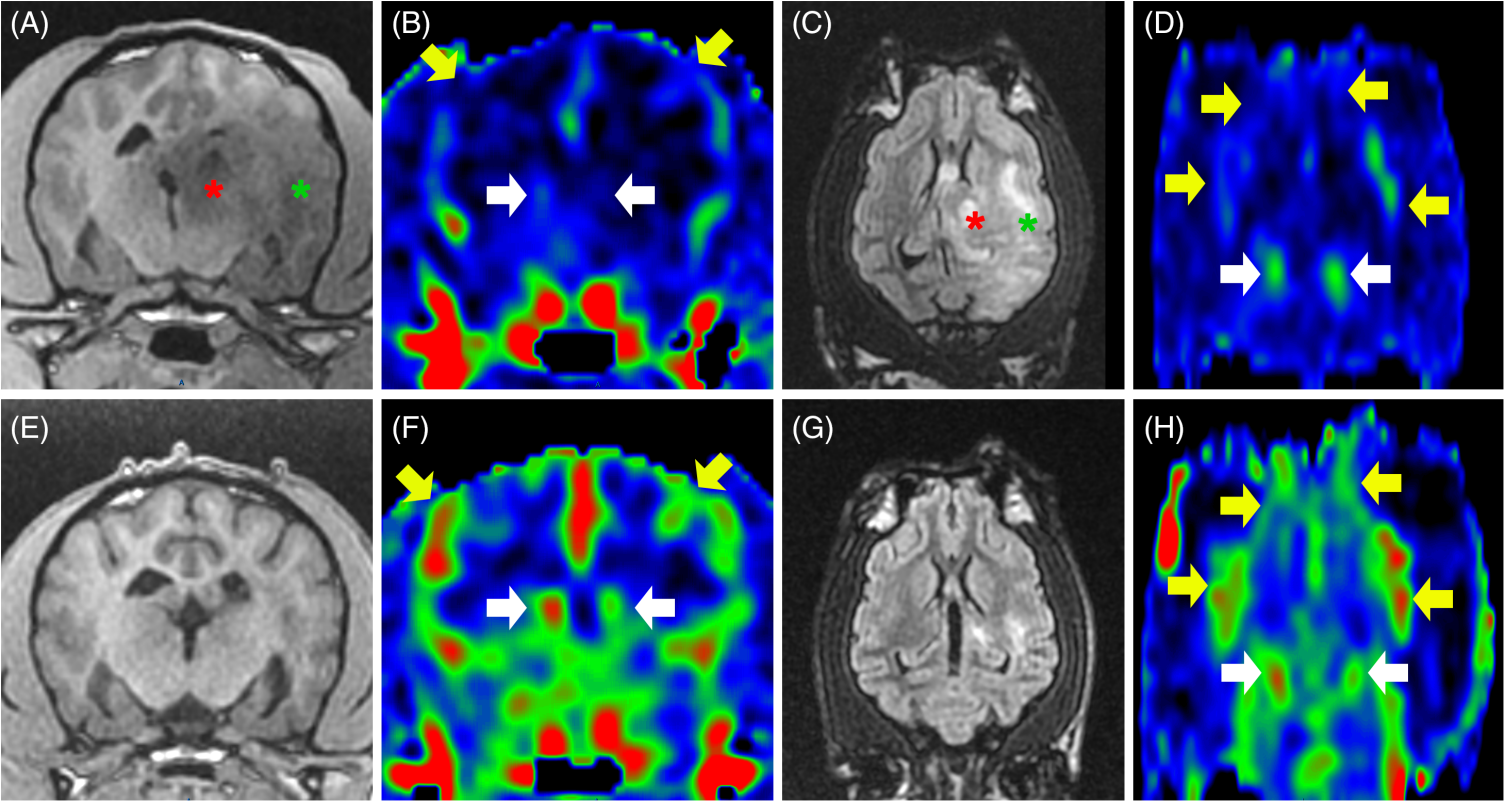
MRI in a 9‐year‐old male Maltese with a diagnosis of MUO, at disease onset (A‐D) and after 6 months of medical treatment (E‐H), including transverse T1W images (A, E) and ASL CBF maps (B, F) at the level of the cranial mesencephalon, and dorsal FLAIR images (C, G) and reconstructed dorsal ASL CBF maps (D, H) at the level of the mesencephalic aqueduct. All ASL CBF maps (B, D, F, H) are displayed with the same window settings (level, 40 mL/100 g/min; width, 70 mL/100 g/min). (A, C) At disease onset, there are large ill‐defined heterogeneous T1W hypointense and FLAIR hyperintense lesions within the left forebrain (green asterisks) and midbrain (red asterisks) associated with features of increased intracranial pressure (left lateral ventricle compression, rightwards midline shift, cortical sulci collapse bilaterally). (B, D) Global brain hypoperfusion indicated by a low ASL signal in the parietal, frontal and temporal cortex (yellow arrows) and thalamico‐mesencephalic nuclei (white arrows) bilaterally. (E, G) After 6 months of medical treatment, the left‐sided forebrain and midbrain lesions have nearly resolved and all features of increased intracranial pressure are no longer present. (F, H) Marked increase in global brain perfusion compared with the pre‐treatment ASL perfusion study (B, D). Mean absolute CBF measured in different normal‐appearing brain regions (white and yellow arrows) has increased by 50% to 170% after treatment. Note that all images obtained in a transverse or dorsal plane are displayed with the right side of the dog on the left side of the image and the left side of the dog on the right side of the image. ASL, arterial spin labeling; CBF, cerebral blood flow; FLAIR, fluid‐attenuated inversion recovery; MRI, magnetic resonance imaging; MUO, meningoencephalitis of unknown origin; T1W, T1‐weighted.

**FIGURE 5:**
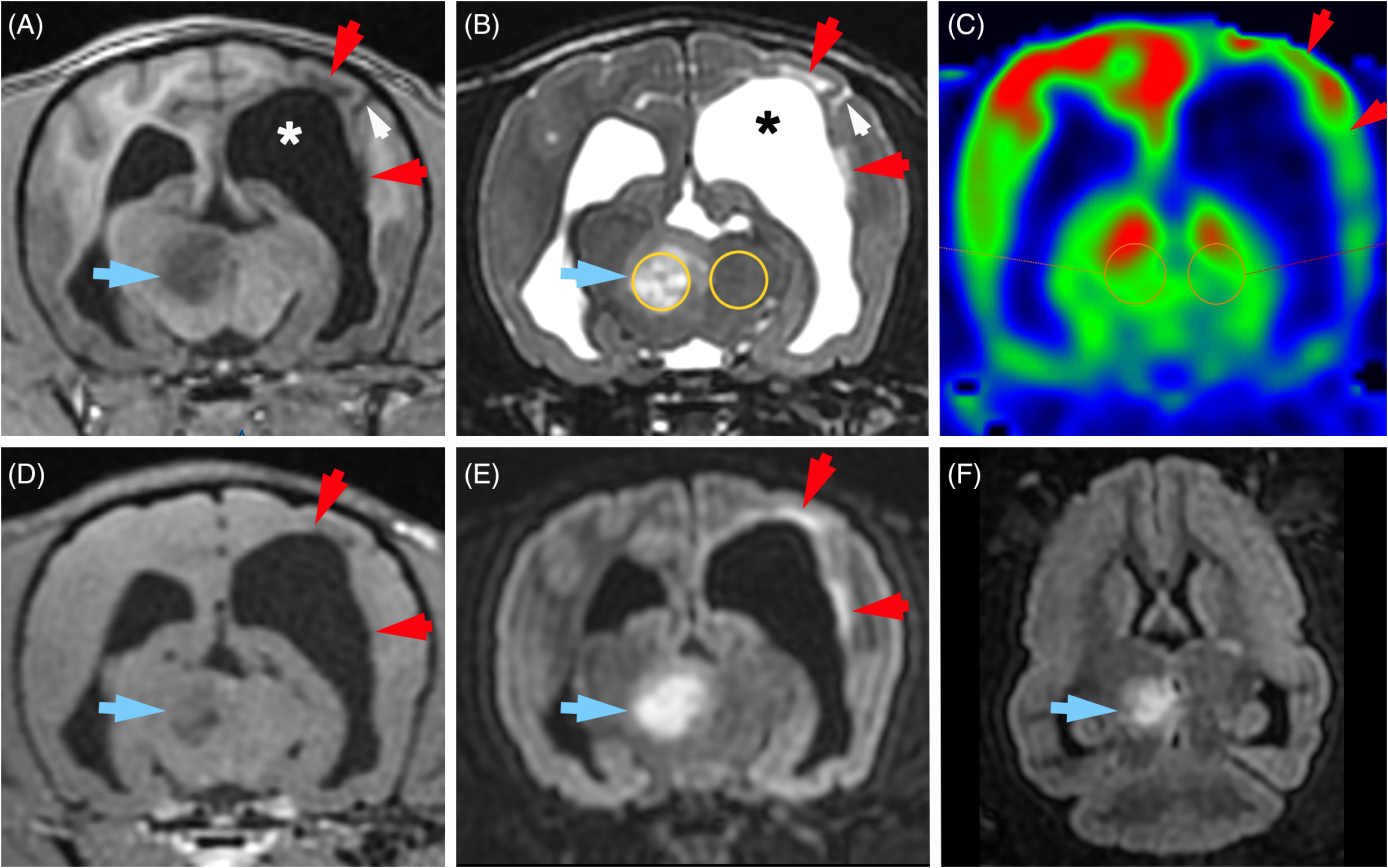
Transverse (A‐E) and dorsal (F) MR images at the level of midbrain, in a 5‐year‐old male Yorkshire terrier with a diagnosis of MUO. There is a heterogeneous T1W (A) hypointense, T2W (B) and FLAIR (E, F) hyperintense nodular lesion within the right mesencephalon (blue arrows) showing faint heterogeneous contrast enhancement (D) and no ASL perfusion changes (C). ROI drawn over the lesion and contralateral parenchyma are indicated by solid circles (B, C). Relative CBF of the lesion is 1.10. There is also a T2W (B) and FLAIR (E) hyperintense, T1W (A) hypointense, not enhancing (D) ill‐defined large area within the periventricular white matter of the left parietal lobe (red arrows). Left‐sided ventricular enlargement (asterisks), focal widening of the left suprasylvian sulcus (white arrow), thining of the left parietal cortex and mild leftwards shift of dorsal falx most likely indicate brain parenchyma loss in the left parietal lobe, suggesting an old brain lesion (atrophy). The left parietal lobe parenchyma also shows a decreased ASL signal intensity compared with the contralateral lobe (C, red arrows). Because of its atrophy pattern, this left parietal lesion has been excluded from ASL perfusion analysis. Note that all images obtained in a transverse or dorsal plane are displayed with the right side of the dog on the left side of the image and the left side of the dog on the right side of the image. ASL, arterial spin labeling; CBF, cerebral blood flow; FLAIR, fluid‐attenuated inversion recovery; MR, magnetic resonance; MUO, meningoencephalitis of unknown origin; ROI, region of interest; T1W, T1‐weighted; T2W, T2‐weighted.

The remaining 3 dogs demonstrated lack of perfusion signal in the whole brain with strong signal in the branches of both external carotid arteries (Table 4). These dogs were considered to have increased intracranial pressure (IPP) based on cortical sulci collapse (3/3), lateral ventricles compression (3/3), mesencephalon compression (3/3), midline shift (2/3), transtentorial herniation (2/3), and cerebellar foraminal herniation (1/3; Figure 4). Additionally, 3 lesions with a focal brain atrophy pattern were excluded from ASL assessment (Figure 5).

**TABLE 4:** Absolute cerebral blood flow on ASL‐T0 and ASL‐ctrl in normal‐appearing brain regions in 3 dogs with presumed intracranial hypertension on initial MRI.

Absolute cerebral blood flow	Case 11		Case 19		Case 26		
	T0	T0 + 2.5 months	T0	T0 + 1 month	T0	T0 + 6 months	
mL/100 g/min	CBF		CBF		CBF	CBF	Increase
Cerebral cortex	26	Dead	35	Dead	21	61	190%
Caudate nuclei	42	‐	20	‐	17	40	135%
Thalamic nuclei	35	‐	38	‐	36	62	72%
Mesencephalic nuclei	60	‐	54	‐	19	67	253%
Cerebellar gray matter	45	‐	50	‐	35	72	106%

*Note*: For informational purposes, median absolute CBF (IQR; range) of the cortex and thalamic nuclei measured in 34 dogs with normal standard brain MRI and using a PLD of 1025 ms are provided: CBF (cortex), 97 mL/100 g/min (70‐128; 44‐127); CBF (thalamus), 103 (86‐122; 40‐169).^51^

Abbreviations: ASL, arterial spin labeling; ASL‐ctrl, control ASL performed after at least 3 months of treatment; ASL‐T0, ASL at first presentation; CBF, cerebral blood flow; IQR, interquartile range; MRI, magnetic resonance imaging; PLD, post‐labeling delay.

**FIGURE 6:**
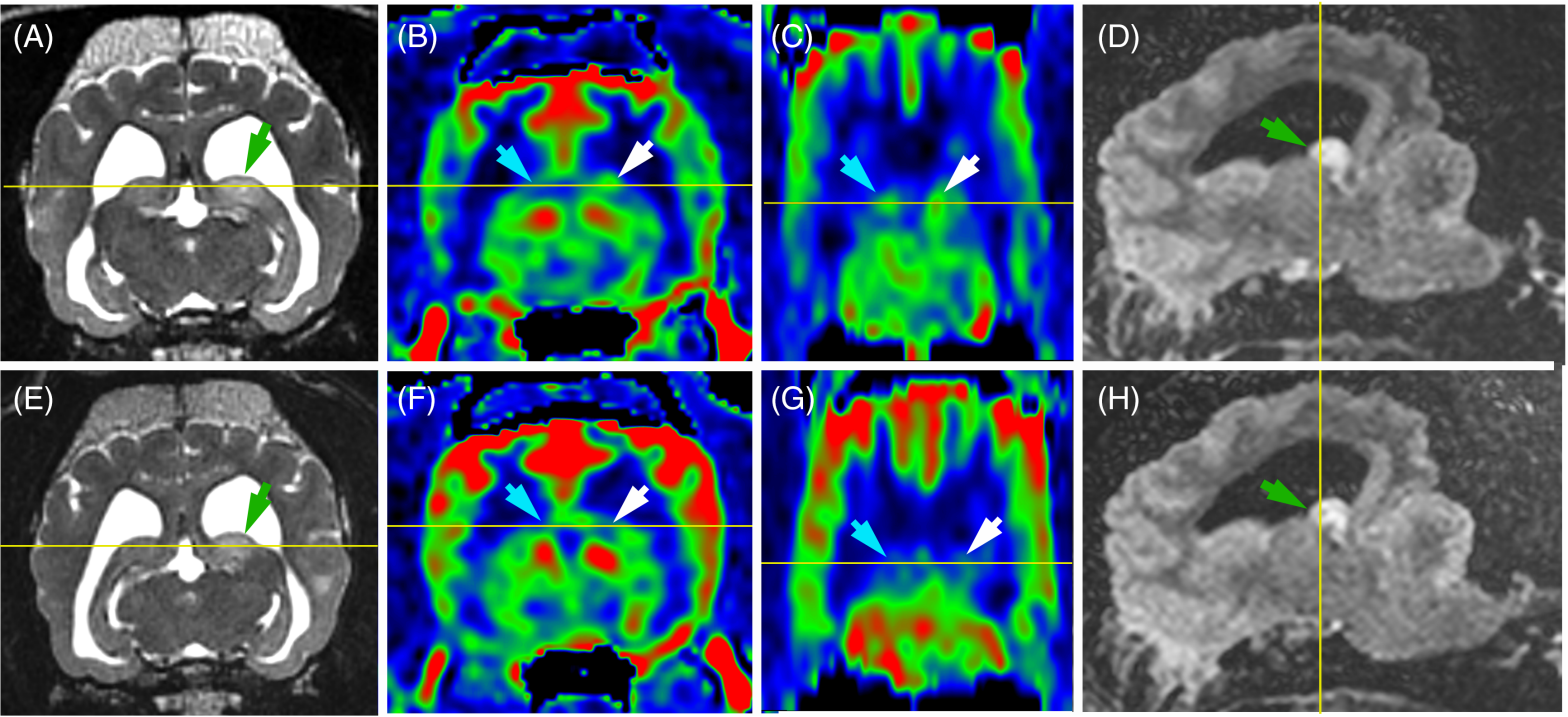
MRI in a 9‐year‐old male French Bulldog with a diagnosis of MUO, at disease onset (A‐D) and after 4 months of medical treatment (E‐H), including transverse T2W images (A, E) and transverse ASL CBF color maps (B, F) at the level of the mesencephalon, and reconstructed dorsal ASL CBF color maps (C, G) and reconstructed sagittal FLAIR images (D, H) at the level of the left dorsal hippocampus. All ASL CBF maps (B, C, F, G) are displayed with the same window settings (level, 72 mL/100 g/min; width, 65 mL/100 g/min). At disease onset, there is a focal T2W (A) and FLAIR (D) hyperintense lesion in the dorsal region of the left hippocampus (green arrows). This lesion shows a focal hyperintensity on ASL perfusion images (B, C, white arrows) as compared with the contralateral area (blue arrows), with a rCBF of 1.35. Solid lines in each image indicate the location of orthogonal images crossing the dorsal left hypocampus. After medical treatment the lesion in the left hippocampus is still present and unchanged on T2W (E) and FLAIR (H) images but does not show any perfusion signal changes on ASL CBF maps (F, G, white arrows) as compared with the contralateral area (blue arrows), and has a rCBF of 0.98. Note that there is a moderate increase in ASL perfusion signal intensity in all cerebral and cerebellar lesion‐free cortical areas after treatment (F, G) compared with pre‐treatment CBF color maps (B, C). After treatment, mean CBF has increased in all assessed regions of disease‐free gray matter by 16% to 48%. Note that all images obtained in a transverse or dorsal plane are displayed with the right side of the dog on the left side of the image and the left side of the dog on the right side of the image. ASL, arterial spin labeling; CBF, cerebral blood flow; FLAIR, fluid‐attenuated inversion recovery; MRI, magnetic resonance imaging; MUO, meningoencephalitis of unknown origin; rCBF, relative cerebral blood flow; T2W, T2‐weighted.

Signal intensity of brain lesions at disease onset was assessed on conventional MRI and ASL sequences in 21 dogs that showed 56 lesions (Figure 1). The prevalence of the different signal intensities on conventional MRI sequences in the 21 dogs (Table 5) were comparable to those noted in the 29 dogs (Table 3). Focal ASL perfusion changes were detected in 9/56 (16%) lesions, corresponding to 8/21 (38%) dogs (Figure 6). Hyperperfusion was noted visually more frequently than hypoperfusion (6/56 [11%] and 3/56 [5%], respectively). One dog had a mixed pattern with 1 hyperperfused lesion and 1 hypoperfused lesion. Three dogs had a single lesion, 2 dogs with hyperperfusion pattern and 1 dog with a hypoperfusion pattern. The remaining 4 dogs had multiple lesions with only 1 lesion showing perfusion changes in each dog. The rCBF was significantly higher in the 6 lesions with hyperperfusion pattern than in the 47 lesions showing no perfusion changes (median [range], 1.44 [1.25‐1.65]; 1 [0.89‐1.14], respectively; Wilcoxon rank‐sum test, *P* < .001).

**TABLE 5:** Brain lesions signal intensityon initial conventional MRI and ASL perfusion in 21 dogs with MUO according to Figure 1.

	Lesions *n* = 56	Dogs *n* = 21
T2W hyperintense	55/56 (98%)	21/21 (100%)
T2W isointense	1/56 (2%)	1/21 (5%)
T2W hypointense	0/56 (0%)	0/21 (0%)
FLAIR hyperintense	54/56 (96%)	21/21 (100%)
FLAIR isointense	2/56 (4%)	2/21 (10%)
FLAIR hypointense	0/56 (0%)	0/21 (0%)
T1W hyperintense	0/56 (0%)	0/21 (0%)
T1W isointense	9/56 (16%)	8/21 (38%)
T1W hypointense	47/56 (84%)	19/21 (90%)
SWI hypointense	0/56 (0%)	0/21 (0%)
Diffusion hyperintense	21/54 (39%)	11/20 (55%)
Diffusion isointense	30/54 (56%)	11/20 (55%)
Diffusion hypointense	3/54 (6%)	1/20 (5%)
Diffusion missing data	2/56	1/21
ADC values increased	45/54 (83%)	20/20 (100%)
ADC values not modified	8/54 (15%)	5/20 (25%)
ADC values decreased	1/54 (2%)	1/20 (5%)
ADC values missing data	2/56	1/21
ASL hyperintense	6/56 (11%)	6/21 (29%)
ASL isointense	47/56 (84%)	17/21 (81%)
ASL hypointense	3/56 (5%)	3/21 (14%)

Abbreviations: ADC, apparent diffusion coefficient; ASL, arterial spin labeling; FLAIR, fluid‐attenuated inversion recovery; MRI, magnetic resonance imaging; MUO, meningoencephalitis of unknown origin; SWI, susceptibility‐weighted imaging; T1W, T1‐weighted; T2W, T2‐weighted.

### 3.5 Clinical and CSF follow‐up after treatment in 23 dogs with MUO

Eight of 31 (26%) dogs died before the MRI‐ctrl, between 4 days and 2 months after treatment initiation (Figure 1). One dog died spontaneously presumably secondary to its neurologic deficits and in the remaining 7 dogs euthanasia was requested by owners because of poor condition and absence of improvement with treatment, and all owners declined necropsy. In the remaining 23/31 dogs, the MRI‐ctrl was performed 3 months (15/23), 4 months (4/23), 5 months, 6 months, 9 months, and 10 months (1/23) after MRI‐T0 with no treatment interruption. At the time of MRI‐ctrl, complete clinical remission was noted in 15/23 (65%) dogs, partial regression in 7/23 (30%) and stable condition in 1/23 (4%), with no surviving dogs showing worsening of neurological signs. Most of the dogs (22/23) were followed after their MRI‐ctrl, over 3 to 12 months for 4/23 (17%), 13 to 24 months for 11/23 (48%) and 25 to 37 months for 7/23 (30%), with no deaths noted, complete remission of neurological signs in 15/23 (65%), partial regression in 6/23 (26%) and relapse after a remission phase and treatment interruption in 2/23 (9%).

At the time of MRI‐ctrl, the initial pleocytosis had resolved in 13/23 (57%) dogs, a pleocytosis was still present in 7/23 (30%) dogs with marked decrease (>60%) in TNCC in 6/7 dogs, and CSF was not re‐evaluated in 3/23 dogs because no pleocytosis had been noted initially.

### 3.6 Follow‐up conventional MRI after treatment in 23 dogs with MUO

The 2 dogs with no lesions on conventional MRI‐T0 had good clinical response to treatment and showed no lesions on MRI‐ctrl. The remaining 21 dogs had 58 lesions on MRI‐T0 that demonstrated either complete remission (19/58 [33%] lesions, 10/21 dogs), partial regression (18/58 [31%] lesions, 12/21 dogs), or stable condition (21/58 [36%] lesions, 8/21 dogs). No dogs showed progression of any lesion either in size or in number.

### 3.7 Follow‐up of lesions on conventional MRI and ASL perfusion after treatment in 12 dogs with MUO

Nine of the 21 surviving dogs with initial brain lesions were excluded from ASL follow‐up study (Figure 1) because of chip‐related labeling artifact either on ASL‐T0 or on ASL‐ctrl. As previously, the 3 lesions showing an atrophy pattern were excluded from ASL‐ctrl assessment. The remaining 12/21 dogs had 27 lesions on MRI‐T0.

On conventional MRI‐ctrl, brain lesions showed either complete remission (9/27, 33%), partial regression (12/27, 44%), or stable condition (6/27, 22%; Table 6). One dog had signs of intracranial hypertension on conventional MRI‐T0 and low global brain perfusion signal on ASL‐T0. After 6 months of treatment, this dog had complete clinical remission, complete remission of MRI features of intracranial hypertension, partial regression of focal brain lesions on conventional MRI, decrease in CSF TNCC (8 cell/μL vs 26 initially) and marked increase in global brain perfusion on ASL‐ctrl (Table 4 [case 26]; Figure 4). In the remaining 11 dogs, all initial focal perfusion abnormalities (6/6) had resolved (Table 7; Figure 6). On conventional MRI, these lesions showed complete remission (2/6), partial regression (3/6) or stable condition (1/6). No de novo perfusion abnormalities were found on ASL‐ctrl in any of the dogs.

**TABLE 6:** Follow‐up of lesions on conventional MRI and ASL perfusion after treatment in 12 dogs with MUO.

	Lesions, n = 27		Dogs, n = 12	
Hypoperfused hemisphere on ASL‐T0 (raised ICP)	2/27	7%	1/12	8%
Normalization of brain perfusion pattern on ASL‐ctrl	2/2	100%	1/1	100%
Hyperperfused focal brain lesions on ASL‐T0	3/27	11%	3/12	25%
Normalization of perfusion pattern on ASL‐ctrl	3/3	100%	3/3	100%
Complete remission on conventional MRI‐ctrl	1/3	33%	1/3	33%
Partial regression on conventional MRI‐ctrl	1/3	33%	1/3	33%
Stable condition on conventional MRI‐ctrl	1/3	33%	1/3	33%
Hypoperfused focal brain lesions on ASL‐T0	3/27	11%	3/12	25%
Normalization of perfusion pattern on ASL‐ctrl	3/3	100%	3/3	100%
Complete remission on conventional MRI‐ctrl	1/3	33%	1/3	33%
Partial regression on conventional MRI‐ctrl	2/3	67%	2/3	67%
Stable condition on conventional MRI‐ctrl	0/3	0%	0/3	0%
Isoperfused focal brain lesions on ASL‐T0	19/27	70%	9/12	75%
Unchanged normal perfusion pattern on ASL‐ctrl	19/19	100%	9/9	100%
Complete remission on conventional MRI‐ctrl	5/19	26%	3/9	33%
Partial regression on conventional MRI‐ctrl	9/19	47%	6/9	67%
Stable condition on conventional MRI‐ctrl	5/19	26%	3/9	33%

Abbreviations: ASL, arterial spin labeling; ASL‐ctrl, ASL performed during a control MRI after at least 3 months of treatment; ASL‐T0, ASL performed at disease onset; CBF, cerebral blood flow; ICP, intracranial pressure; MRI, magnetic resonance imaging; MRI‐ctrl, control MRI.

**TABLE 7:** Follow‐up ASL‐MRI of 9 brain lesions showing initial focal perfusion alterations.

Case	Location of lesion	Brain perfusion ASL‐T0			Delay between MRI‐T0 and MRI‐ctrl (months)	Brain perfusion ASL‐ctrl		
		Pattern on visual assessment	Mean absolute CBF values (mL/100 mg/min)	Mean rCBF values (unitless)		Pattern on visual assessment	Mean absolute CBF values (mL/100 mg/min)	Mean rCBF values (unitless)
3	Optic chiasm	Focal hyperperfusion	70	1.52	4	Normalized perfusion signal	48	0.93
4	Hippocampus	Focal hyperperfusion	68	1.36	4	Normalized perfusion signal	59	1
28	Brain stem	Focal hyperperfusion	107	1.32	3	Normalized perfusion signal	93	0.95
25	Brain stem	Focal hyperperfusion	66	1.55	Labeling artifact	ND	ND	ND
9	Thalamus	Focal hyperperfusion	120	1.25	Labeling artifact	ND	ND	ND
8	Cerebellum	Focal hyperperfusion	191	1.55	Dead before MRI‐ctrl	ND	ND	ND
3	Thalamus	Focal hypoperfusion	17	0.34	4	Normalized perfusion signal	78	1.03
27	Cortex and adjacent WM	Focal hypoperfusion	29	0.66	9	Normalized perfusion signal	41	0.95
29	Thalamus	Focal hypoperfusion	32	0.71	3	Normalized perfusion signal	81	1.08

Abbreviations: ASL, arterial spin labeling; ASL‐ctrl, control ASL; ASL‐T0, ASL at disease onset; CBF, cerebral blood flow; MRI, magnetic resonance imaging; MRI‐ctrl, control MRI; MRI‐T0, MRI at disease onset; ND, not determined; rCBF, relative CBF; WM, white matter.

### 3.8 Follow‐up of global brain ASL perfusion in 12 dogs with MUO and initial brain lesions

**FIGURE 7:**
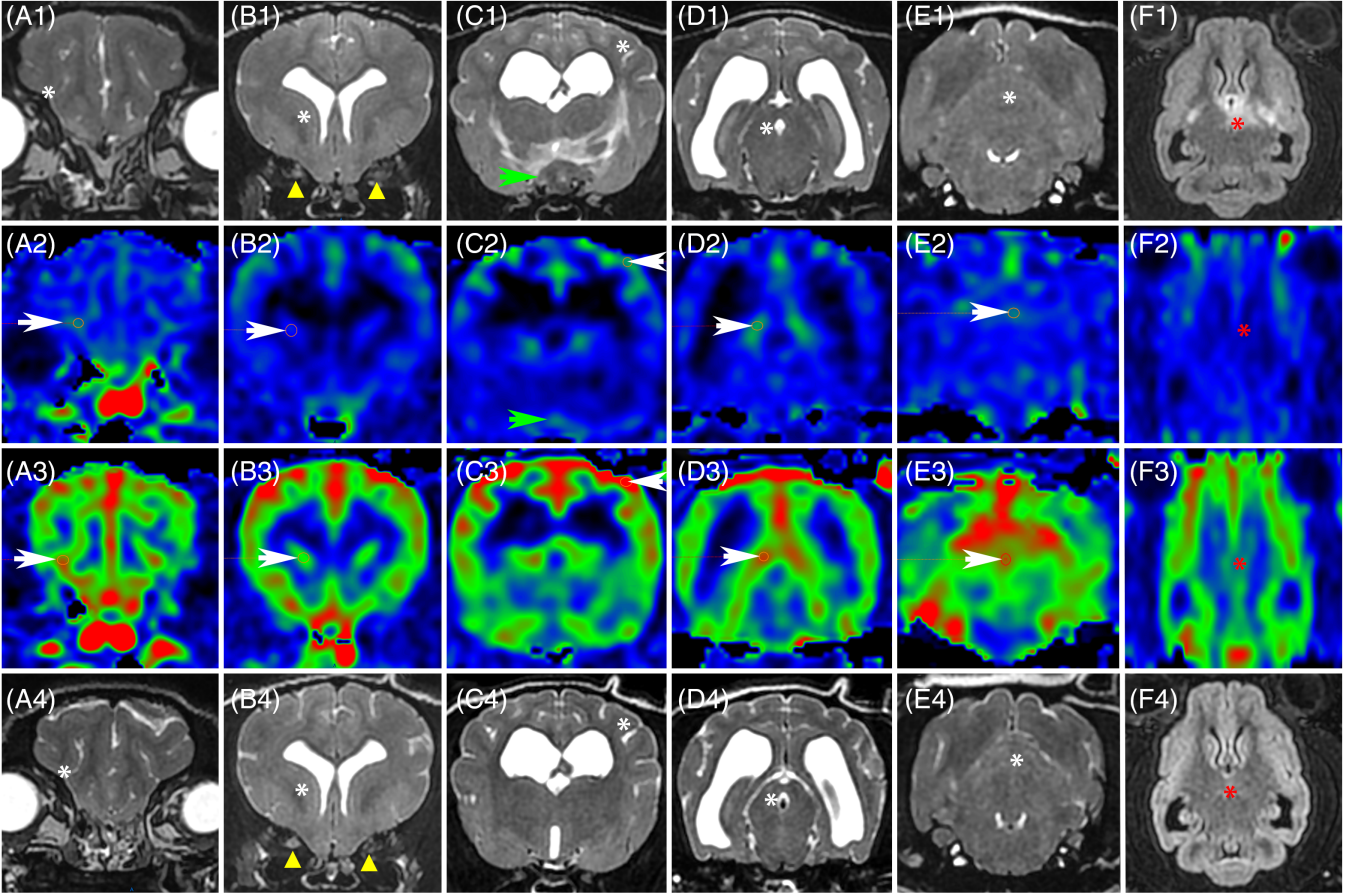
MRI in a 4‐year‐old male Brabançon with a diagnosis of MUO, at disease onset (A1‐F1, A2‐F2) and after 4 months of medical treatment (A3‐F3, A4‐F4), including transverse T2W images (A1‐E1, A4‐E4) and transverse ASL CBF color maps (A2‐E2, A3‐E3) covering the brain, and dorsal FLAIR images (F1, F4) and reconstructed dorsal ASL CBF color maps (F2, F3) at the level of the interthalamic adhesion (red asterisks). All ASL CBF color maps (A2‐F2, A3‐F3) are displayed with the same window settings (level, 63 mL/100 g/min; width, 112 mL/100 g/min). At disease onset, there is a T2W slightly hypointense 8 mm‐wide mass at the optic chiasm location (C1, green arrow) that is compressing and dorsally displacing the ventral part of the 3rd ventricle, and shows no ASL perfusion signal changes compared with adjacent parenchyma (C2, green arrow). Dorsal to the mass, an ill‐defined T2W (C1) and FLAIR (F1) hyperintense area affecting the ventral thalamus and left internal capsule is noted. Note the 2 optic nerves (B1, yellow arrow heads) that do not show the normal T2W hyperintense peripheral ring of CSF, suggesting optic nerves swelling. A low ASL perfusion signal within the entire brain (A2‐F2) is present. After 4 months of medical treatment, the optic chiasm mass and the T2W hyperintensity in the ventral thalamus and left internal capsule have resolved (C4, F4), and a marked increase in global brain perfusion (A3‐F3) is noted compared with the pre‐treatment ASL perfusion study (A2‐F2). Mean absolute CBF measured in different normal‐appearing brain regions (white arrows and asterisks) has increased by 70% to 120%. Note that the right optic nerve (B4, yellow arrow head) displays a peripheral T2W hyperintense ring of CSF consistent with a reduced swelling of the nerve, while the left optic nerve is unchanged compared with pre‐treatment image (B1). Note that all images obtained in a transverse or dorsal plane are displayed with the right side of the dog on the left side of the image and the left side of the dog on the right side of the image. ASL, arterial spin labeling; CBF, cerebral blood flow; CSF, cerebrospinal fluid; FLAIR, fluid‐attenuated inversion recovery; MRI, magnetic resonance imaging; MUO, meningoencephalitis of unknown origin; T2W, T2‐weighted.

In the 12 dogs with initial brain lesions and diagnostic quality ASL‐T0 and ASL‐ctrl, the median time between MRI‐T0 and MRI‐ctrl was 3.5 months (IQR, 3‐6; range, 3‐10). In this group of dogs, each assessed lesion‐free brain region showed a significant increase (Wilcoxon signed‐rank test, *P* < .05) in absolute CBF after treatment (Table 8). In 4/12 dogs, the increase in CBF was <15% in almost all regions. In the remaining 8/12 dogs, a substantial increase in CBF was observed in all regions with a median increase of 47% (IQR, 27%‐109%; range, 18%‐253%; Figure 7).

**TABLE 8:** Global brain ASL perfusion (lesion‐free brain regions) in 12 dogs with MUO and initial brain lesions, at disease onset and after treatment according to Figure 1.

Median CBF (IQR; range), mL/100 g/min	At disease onset	After treatment of ≥3 months duration	*P*‐value
Cerebral cortex n = 12	78 (54‐97; 21‐105)	98 (82‐139; 61‐153)	<.01
Caudate nuclei n = 12	58 (40‐71; 17‐93)	72 (66‐97; 40‐117)	<.01
Thalamic nuclei n = 12	78 (52‐97; 36‐113)	92 (77‐115; 62‐139)	<.05
Mesencephalic nuclei n = 11	98 (67‐104; 19‐123)	111 (95‐145; 67‐147)	<.01
Cerebellar cortex n = 11	81 (41‐87; 35‐101)	101 (77‐118; 63‐135)	<.01

*Note*: For informational purposes, median absolute CBF (IQR; range) of the cortex and thalamic nuclei measured in 34 dogs with normal standard brain MRI and using a PLD of 1025 ms are provided: CBF (cortex), 97 mL/100 g/min (70‐128; 44‐127); CBF (thalamus), 103 (86‐122; 40‐169).^51^ Differences of ASL perfusion between disease onset and after treatment were tested by Wilcoxon signed‐rank test. Differences were considered significant for *P*‐value <.05.

Abbreviations: ASL, arterial spin labeling; CBF, cerebral blood flow; IQR, interquartile range; MRI, magnetic resonance imaging; MUO, meningoencephalitis of unknown origin; PLD, post‐labeling delay.

### 3.9 Focal ASL perfusion changes, seizures, and death

The mortality rate was not significantly different between the 6/23 dogs with hyperperfused lesions and the 17/23 dogs that did not show focal hyperperfusion (1/6 [16.7%] and 3/17 [17.6%], respectively; Fisher's exact test, *P* = 1) and was below the mortality rate in the entire population (8/31, 26%).

Premature death, during the first 2 months of treatment, was observed in 4/6 (67%) dogs with initial seizures and in 4/25 (16%) dogs without initial seizures. A significant association was found between the occurrence of seizures before treatment and premature death (Fisher's exact test, *P* < .05).

No significant association was found between the presence of a hyperperfused brain lesion and the occurrence of seizures (Fisher's exact test, *P* = .34).

## 4 DISCUSSION

We describe the brain signal changes on conventional MRI and 3D‐pCASL perfusion in 31 dogs with MUO at disease onset and after several months of medical treatment.

### 4.1 Clinical findings

In the study population, middle‐aged toy and small breed dogs were overrepresented, consistent with the literature.^5^, ^17^, ^18^, ^52^, ^53^, ^54^, ^55^ The female : male ratio was slightly <1, which differs from previous studies in which a higher risk of developing the condition was reported in females.^4^, ^5^, ^8^, ^17^, ^56^, ^57^ As reported previously, all deaths occurred early (within the first 2 months of treatment), epilepsy was a significant risk factor for a premature death, and dogs without detectable lesions on conventional MRI seemed to have better outcome than dogs with detectable lesions.^8^, ^17^, ^19^, ^53^, ^58^, ^59^, ^60^

### 4.2 Conventional MRI at disease onset in dogs with MUO

Similarly to previous studies on MUO, few dogs in the study population (2/31, 6%) had normal brain MRI, and in dogs with brain anomalies the most common finding was multifocal asymmetrical brain lesions.^2^, ^4^ A single focal lesion has been reported in dogs with the focal form of GME, but the prevalence of a single brain MRI lesion in MUO has not been clearly documented.^1^, ^5^ We report here 19% (6/31) of dogs with MUO with a single brain lesion on MRI. Symmetrical multiple brain lesions, suggesting subacute necrotizing meningoencephalitis, also were observed but with lower frequency (6%, 2/31).^10^ This result indicates that atypical brain lesion distribution (ie, single lesion and symmetrical multiple lesions) may not be uncommon (25% in our study). In such cases, MRI features of MUO overlap those of other brain diseases including glioma, toxic‐metabolic encephalopathy, infectious granulomatous encephalitis and ischemic stroke, making differential diagnosis difficult.

The telencephalon was the most commonly affected area (69% of the dogs with detectable lesions), followed by the brainstem (55%), diencephalon (34%), and cerebellum (24%). This result is consistent with the strong predilection of lesions for the cerebral hemispheres and the weakest predilection for the cerebellum previously reported in dogs with GME, NLE, and NME.^7^, ^10^, ^14^, ^61^ However, in previous studies, the brainstem was less frequently involved than the diencephalon, in contrast to our observations.

A substantial proportion of dogs (12/31, 39%) had ON involvement on FLAIR or contrast T1W sequences or both. Ocular lesions in MUO have been reported mostly in the ocular form of GME.^1^, ^12^, ^15^ In 12/12 dogs, lesions in the retrobulbar segment of 1 or both ONs were detected on MRI. Eleven of these dogs had a follow‐up and 8/11 (73%) exhibited a return to normal vision after medical treatment. In 2/12 dogs, a mass in the optic chiasm area was found on MRI‐T0 that had resolved after 3 and 4.5 months of treatment. The good therapeutic response and long survival times observed in the dogs with ON or optic chiasm lesions are not consistent with the poor prognosis previously reported in the ocular form of GME, illustrating that ON involvement might be encountered in other types of MUO than the ocular form of GME.^5^, ^12^

In our study, dogs with MUO also showed preferential localization of lesions in ONs or brainstem or both, with involvement of ONs in 39% (12/31) of dogs, brainstem in 52% (16/31) and 1 or 2 of these regions in 68% (21/31). In humans, localization of lesions in ONs or brainstem have been reported in myelin oligodendrocyte glycoprotein antibody‐associated disease (MOGAD) as distinct MRI features allowing distinction of this encephalitis from other acquired CNS demyelinating diseases such as multiple sclerosis and aquaporin‐4‐seropositive neuromyelitis optica spectrum disorder.^45^, ^49^, ^62^ This similarity in lesion localization between some dogs with MUO and humans with MOGAD may represent a new argument for the existence of blood biomarkers specific for some MUO in dogs, as in humans, that remain to be identified by antibody research.^12^, ^45^, ^49^, ^62^, ^63^

The brain lesions' signal intensity and enhancement pattern in our study were in agreement with previous reports.^1^, ^2^, ^10^ Only 1/82 lesions showed a T2W hyperintensity that was partially suppressed on FLAIR, suggesting a liquid‐like texture consistent with advanced necrosis and detectable cavitation.

The MRI features of MUO in dogs have not yet been described on SWI and DWI in the current literature. In our study, none of the 82 lesions had SWI hypointensity, which is consistent with the absence of a hemorrhagic component as reported in previous histopathological studies.^3^, ^5^, ^12^, ^14^ Only 9/80 (11%) lesions had a DWI hyperintense signal with decreased ADC value, indicating diffusion restriction suggestive of cytotoxic edema. Five of these lesions were observed in the same dog that was euthanized 4 days after MRI‐T0, and the 4 remaining lesions were seen in 4 different dogs that survived at least 6 months with partial or complete clinical remission. This result suggests that a restriction of diffusion of brain lesions might not be correlated with outcome in dogs with MUO.

### 4.3 Failure of ASL perfusion sequence in dogs with MUO

Poor ASL quality because of a microchip‐related labeling failure artifact was noted at MRI‐T0 and MRI‐ctrl (5/31 [16%] and 9/23 [39%] dogs, respectively). These ASL failure rates were much higher than the rate of 4% previously reported in a group of 74 dogs with higher weights.^51^ This study concluded that the lighter the dogs (<6.5 kg) and the shorter the chip‐to‐foramen magnum distance (<6 cm), the higher the risk of chip‐related labeling artifact. The relatively high rates of labeling failure artifact in our study may reflect selection bias, because MUO is mostly encountered in small breed dogs.

### 4.4 ASL perfusion in dogs with MUO and presumed intracranial hypertension

A low global brain perfusion signal was visually noted in 3/29 dogs at disease onset whereas both carotid arteries had a normal perfusion signal, indicating proper arterial blood tagging. In humans, diffusely decreased CBF on ASL perfusion imaging is associated with a broad differential diagnosis, including increased arterial transit time of magnetically tagged protons, brain atrophy, vasculitis and cerebral vasospasm caused by exogenous agents or subarachnoid hemorrhage, encephalomalacia, childhood encephalitis, and impaired consciousness.^24^, ^37^, ^64^ In each of the 3 dogs, increased intracranial pressure (IIP) could have been a potential cause of global brain hypoperfusion. This suspicion was reinforced by the substantial increase in CBF (from 72% to 253% depending on the assessed regions) after treatment in the dog that survived and showed complete remission of neurological signs, complete remission of MRI features suggesting IIP, and partial regression of brain lesions on conventional MRI. This result suggests that ASL may have added value in diagnosing and monitoring intracranial hypertension.

### 4.5 Initial and post‐treatment ASL perfusion of focal brain lesions in dogs with MUO

The perfusion of focal brain lesions at disease onset was assessed in 21 dogs. As reported in encephalitides of humans, hyperperfusion pattern was more commonly observed than hypoperfusion or mixed pattern (5/8 dogs, 2/8 and 1/8, respectively).^24^, ^38^, ^39^, ^40^, ^41^, ^42^, ^44^ The mechanism of perfusion changes has not been fully elucidated and may vary according to the underlying cause (infectious, autoimmune, inflammatory demyelinating) and evolves according to the stage of the disease. In the acute phase, vasculitis may lead to vasodilatation which, in turn, would increase metabolism and local CBF.^33^, ^34^, ^43^, ^64^, ^65^ In a more chronic phase, focal hypoperfusion may result from excessive neuronal damage and loss of brain parenchyma. Similarly, brain areas of hypometabolism, most likely representing altered neuronal function, have been observed in autoimmune encephalitis in humans using nuclear medicine and may explain the hypoperfusion pattern noted on ASL.^43^, ^66^

In our study, focal brain perfusion changes were noted in only 38% (8/21) of the dogs whereas conventional MRI abnormalities suggesting meningoencephalitis were seen in 94% (29/31). Additionally, no obvious ASL perfusion changes were noted on visual assessment in the normal‐appearing brain areas in the 21 dogs with brain lesions and in the 2 dogs with normal conventional MRI. This result differs markedly from several small case series of humans with autoimmune encephalitis, in which increased CBF was noted in affected areas in all patients, and in normal or slightly abnormal brain areas on conventional MRI, suggesting that ASL could improve the sensitivity of lesion detection.^38^, ^39^, ^40^, ^41^, ^42^, ^44^ The reason for this discrepancy is unknown but might be related to different mechanisms involved in autoimmune encephalitis in humans and in dogs with MUO. In our study, ASL did not have higher sensitivity than conventional MRI in the detection of focal brain lesions of MUO in dogs, but the sensitivity of ASL in each subtype of MUO in dogs remains to be determined.

Follow‐up ASL‐MRI after treatment in 12 dogs demonstrated complete disappearance of all focal perfusion changes. A similar observation in humans with encephalitis has been interpreted as a sign of good focal neurologic outcome.^34^ Concomitantly, on conventional MRI only some lesions with perfusion changes showed complete remission whereas others had decreased in size or remained unchanged.

### 4.6 Follow‐up of global brain ASL perfusion in 12 dogs with MUO and initial brain lesions

The absolute CBF in lesions‐free areas in 12 dogs with MUO was significantly higher after treatment than before (Wilcoxon signed‐rank test, *P* < .05), and post‐treatment CBF results were comparable to those previously published in dogs with normal‐appearing brains.^51^ All 12 dogs exhibited improvement of neurological signs and brain lesions on conventional MRI, suggesting that the increase in global brain perfusion reflected initial global hypoperfusion rather than post‐treatment global hyperperfusion. A CBF increase much >15% in all assessed lesion‐free areas was noted in 8/12 (67%) dogs, suggesting that hypoperfusion of the entire brain would be a common pathophysiological component of MUO in dogs. In 7/8 dogs no evidence of intracranial hypertension was found on conventional MRI. It therefore can be speculated that entire brain hypoperfusion would mostly result from diffuse brain damage and hypometabolism, suggesting that ASL could be helpful for monitoring entire brain function in dogs with MUO.

In contrast with our study, global brain hypoperfusion has been associated with poor neurological outcome in childhood encephalitis.^37^ Ten dogs were considered to have global brain hypoperfusion at presentation. Two of these dogs died before their control MRI, giving a mortality rate of 20% in the 10 dogs with initial global brain hypoperfusion. This rate is lower than the mortality rate in the entire study population (26%), suggesting that a global hypoperfusion pattern at disease onset would not permit prediction of poor clinical outcome in dogs with MUO.

### 4.7 Study limitations

Our study had several limitations. Several physiological variables that may influence global brain ASL perfusion signal, such as heart rate, systemic blood pressure, respiratory carbon dioxide concentration, and red blood cell count were not recorded. However, focal brain perfusion changes were identified on the basis of perfusion asymmetry and therefore should not be caused or influenced by these systemic factors.

In dogs with a history of seizures, although no obvious epileptic episode was detected within the 12 hours preceding initial MRI in most dogs, subclinical epileptic activity affecting CBF could not be completely excluded, particularly in the absence of electroencephalography performed just before or during MRI examinations.^35^, ^38^

A major limitation of our study was the lack of histopathological confirmation, which prevented identification of potential differences in perfusion patterns among the different subtypes of MUO, and limited understanding of perfusion changes. Additional studies including longitudinal data and histopathological analysis are required to identify any potential disease‐specific perfusion changes.

Finally, sample size was relatively small and inevitably decreased by technical issues (microchip‐related artifact) and exacerbated by the small size of the dogs. Our study is a first step in identifying CBF changes in MUO in dogs and should be complemented by additional investigations on larger cohorts.

## 5 CONCLUSION

Our prospective study is the first survey on brain perfusion using 3D‐pCASL in dogs with MUO. It determined that, at presentation, one‐third of the dogs with MUO showed focal brain perfusion alteration, and two‐thirds displayed global brain hypoperfusion, but ASL failed to predict poor prognosis. Additionally, our study suggested a potential value of ASL in diagnosing and monitoring intracranial hypertension. Consequently, we recommend adding ASL perfusion in the clinical evaluation of dogs with suspected MUO. Additional studies on larger cohorts and with histopathological confirmation are required for better characterizing the benefit of ASL on diagnosis and follow‐up in the different subtypes of MUO in dogs. Our study also reported preferential lesion localization in the ONs and brainstem, suggesting that ON involvement might be a more common MRI finding than previously reported in dogs with MUO. In humans, similar preferential lesion localization in ON or brainstem or both is strongly suggestive of a specific inflammatory demyelinating antibody‐associated CNS disease, supporting additional studies in antibody research in dogs with MUO. Finally, we showed that a restriction of diffusion in brain lesions of dogs with MUO is infrequently observed and does not seem to be associated with poor outcome.

## CONFLICT OF INTEREST DECLARATION

Authors declare no conflict of interest.

## OFF‐LABEL ANTIMICROBIAL DECLARATION

Authors declare no off‐label use of antimicrobials.

## INSTITUTIONAL ANIMAL CARE AND USE COMMITTEE (IACUC) OR OTHER APPROVAL DECLARATION

Approved by the Ethics Committee Jacques Bonnod of VetAgro Sup, registered #18 by the French Ministry of Research and Education.

## HUMAN ETHICS APPROVAL DECLARATION

Authors declare human ethics approval was not needed for this study.

## Supporting information


**Supporting Information S1:** Implemented conventional MRI sequences.

**Supporting Information S2:** Arterial spin labeling sequence settings.

**Supporting Information S3:** Clinical information of 31 dogs with MUO.

## References

[1] Coates JR , Jeffery ND . Perspectives on meningoencephalomyelitis of unknown origin. Vet Clin Small Anim. 2014;44:1157‐1185. 10.1016/j.cvsm.2014.07.009 25239815

[2] Cornelis I , Van Ham L , Gielen I , et al. Clinical presentation, diagnostic findings, prognostic factors, treatment and outcome in dogs with meningoencephalomyelitis of unknown origin: a review. Vet J. 2016;244:37‐44. 10.1016/j.tvjl.2018.12.007 30825893

[3] Uchida K , Park E , Tsuboi M , Chambers JK , Nakayama H . Pathological and immunological features of canine necrotising meningoencephalitis and granulomatous meningoencephalitis. Vet J. 2016;213:72‐77. doi:10.1016/j.tvjl.2016.05.002 27240919

[4] Granger N , Smith PM , Jeffery ND . Clinical findings and treatment of non‐infectious meningoencephalomyelitis in dogs: a systematic review of 457 published cases from 1962 to 2008. Vet J. 2010;184:290‐297. 19410487 10.1016/j.tvjl.2009.03.031

[5] Talarico LR , Schatzberg SJ . Idiopathic granulomatous and necrotising inflammatory disorders of the canine central nervous system: a review and future perspectives. J Small Anim Pract. 2010;51:138‐149. doi:10.1111/j.1748-5827.2009.00823x 19814766

[6] Lobetti RG , Pearson J . Magnetic resonance imaging in the diagnosis of focal granulomatous meningoencephalitis in two dogs. Vet Radiol Ultrasound. 1996;37:424‐427.

[7] Cherubini GB , Platt SR , Anderson TJ , et al. Characteristics of magnetic resonance images of granulomatous meningoencephalomyelitis in 11 dogs. Vet Rec. 2006;159:110‐115. 16861389 10.1136/vr.159.4.110

[8] Cornelis I , Volk HA , de Decker S . Clinical presentation, diagnostic findings and long‐term survival in large breed dogs with meningoencephalitis of unknown aetiology. Vet Rec. 2016;179:147. doi:10.1136/vr.103640 27165997

[9] Flegel T , Henke D , Boettcher IC , et al. Magnetic resonance imaging findings in histologically confirmed Pug dog encephalitis. Vet Radiol Ultrasound. 2008;49:419‐424. 18833947 10.1111/j.1740-8261.2008.00400.x

[10] Flegel T . Breed‐specific magnetic resonance imaging characteristics of necrotizing encephalitis in dogs. Front Vet Sci. 2017;4:1‐7. doi:10.3389/fvets.2017.00203 29255715 PMC5723069

[11] Kaczmarska A , José‐Lopez R , Czopowicz M , et al. Prosencephalic epilepsy in dogs with meningoencephalitis of unknown origin: clinical features, risk factors, and long‐term outcome. J Vet Intern Med. 2020;34:808‐820. doi:10.1111/jvim.15687 31990104 PMC7096646

[12] Kitagawa M , Okada M , Watari T , et al. Ocular granulomatous meningoencephalomyelitis in a dog: magnetic resonance images and clinical findings. J Vet Med Sci. 2009;71:233‐237. 19262040 10.1292/jvms.71.233

[13] Levitin HA , Lampe R , Hecht S . Case report: Meningoencephalomyelitis of unknow etiology manifesting as a bilateral polyneuropathy in 3 dogs. Front Vet Sci. 2020;7:326. doi:10.3389/fvets.2020.00326 32596270 PMC7303259

[14] Young BD , Levine JM , Fosgate GT , et al. Magnetic resonance imaging characteristics of necrotizing meningoencephalitis in pugs dogs. J Vet Intern Med. 2009;23:527‐535. doi:10.1111/j.1939-1676.2009.0306.x 19645838

[15] Muñiz Moris L , Cherubini GB , Caine A . Low‐field magnetic resonance imaging findings in 18 dogs with presumed optic neuritis. Front Vet Sci. 2021;7:585828. 33490127 10.3389/fvets.2020.585828 PMC7817917

[16] Oliphant BJ , Barnes Heller HL , White JM . Retrospective study evaluating associations between midline brain shift on magnetic resonance imaging and survival in dogs diagnosed with meningoencephalitis of unknown etiology. Vet Radiol Ultrasound. 2017;58:38‐43. 27774741 10.1111/vru.12434

[17] Muñana KR , Luttgen PJ . Prognostic factors for dogs with granulomatous meningoencephalomyelitis: 42 cases (1982‐1996). J Am Vet Med Assoc. 1998;212:1902‐1906. 9638190

[18] Coates JR , Barone G , Dewey CW , Vitale CL , Holloway‐Azene NM , Sessions JK . Procarbazine as adjunctive therapy for treatment of dogs with presumptive antemortem diagnosis of granulomatous meningoencephalomyelitis: 21 cases (1998‐2004). J Vet Intern Med. 2007;21:100‐106. 17338156 10.1892/0891-6640(2007)21[100:paatft]2.0.co;2

[19] Lowrie M , Smith PM , Garosi L . Meningoencephalitis of unknown origin: investigation of prognostic factors and outcome using a standard treatment protocol. Vet Rec. 2013;172:527. doi:10.1136/vr.101431 23462382

[20] Alsop DC , Detre JA , Golay X , et al. Recommended implementation of arterial spin‐labeled perfusion MRI for clinical applications: a consensus of the ISMRM Perfusion Study Group and the European Consortium for ASL in Dementia. Magn Reson Med. 2015;73:102‐116. doi:10.1002/mrm.25197 24715426 PMC4190138

[21] Grade M , Hernandez Tamames JA , Pizzini FB , Achten E , Golay X , Smits M . A neuroradiologist's guide to arterial spin labeling MRI in clinical practice. Neuroradiology. 2015;57:1181‐1202. doi:10.1007/s00234-015-1571-z 26351201 PMC4648972

[22] Deibler AR , Pollock JM , Kraft RA , Tan H , Burdette JH , Maldjian JA . Arterial spin‐labeling in routine clinical practice, part 1: technique and artifacts. Am J Neuroradiol. 2008;29:1228‐1234. doi:10.3174/ajnr.A1030 18372417 PMC4686140

[23] Mohindra N , Neyaz Z . Cerebral blood flow measurement with arterial spin labeling MRI: an underutilized technique. Neurol India. 2019;63:834‐836. doi:10.4103/0028-3886.263224 31347563

[24] Bambach S , Smith M , Pezarse Morris P , et al. Arterial spin labeling applications in pediatric and adult neurologic disorders. J Magn Reson Imaging. 2022;53:1‐22. doi:10.1002/jmri.27438 33314349

[25] Blauwblomme T , Boddaert N , Chémaly N , et al. Arterial spin labeling MRI: a step forward in non‐invasive delineation of focal cortical dysplasia in children. Epilepsy Res. 2014;108:1932‐1939. doi:10.1016/j.eplepsyres.2014.09.029 25454505

[26] Blauwblomme T , Naggara O , Brunelle F , et al. Arterial spin labeling magnetic resonance imaging: toward noninvasive diagnosis and follow‐up of pediatric brain arteriovenous malformations. J Neurosurg Pediatr. 2015;15:451‐458. doi:10.3171/2014.9.PEDS14194 25634818

[27] Blauwblomme T , Lemaitre H , Naggara O , et al. Cerebral blood flow improvement after indirect revascularization for pediatric Moyamoya disease: a statistical analysis of arterial spin‐labeling MRI. Am J Neuroradiol. 2016;37:706‐712. doi:10.3174/ajnr.A4592 26585258 PMC7960163

[28] Boulouis G , Shotard E , Dangouloff‐Ros V , et al. Magnetic resonance imaging arterial‐spin‐labeling perfusion alterations in childhood migraine with atypical aura: a case‐control study. Dev Med Child Neurol. 2016;58:965‐969. doi:10.1111/dmcn.13123 27060350

[29] Dangouloff‐Ross V , Grevent D , Pagès M , et al. Choroid plexus neoplasms: toward a distinction between carcinoma and papilloma using arterial spin‐labeling. Am J Neuroradiol. 2015;36:1786‐1790. doi:10.3174/ajnr.A4332 26021621 PMC7968761

[30] Dangouloff‐Ross V , Deroulers C , Foissac F , et al. Arterial spin labeling to predict brain tumor grading in children: correlations between histopathologic vascular density and perfusion MR imaging. Radiology. 2016;281:553‐566. doi:10.1148/radiol.2016152228 27257950

[31] Majer M , Mejdoubi M , Schertz M , Colombani S , Arrigo A . Raw arterial spin labeling data can help identify arterial occlusion in acute ischemic stroke. Stroke. 2015;46:e141‐e144. doi:10.1161/STROKEAHA.114.008496 25931469

[32] Tang S , Liu X , He L , Liu B , Qin B , Feng C . Application of a 3D pseudocontinuous arterial spin‐labeled perfusion MRI scan combined with a post‐labeling delay value in the diagnosis of neonatal hypoxic‐ischemic encephalopathy. PLoS One. 2019;14:e0219284. doi:10.1371/journal.pone.0219284 31283776 PMC6613698

[33] Cao Y , Xiao N , Hu S , Tang Q , Zhou H . Role of magnetic resonance three‐dimensional arterial spin labeling perfusion in diagnosis and follow‐up of viral encephalitis in children. Int J Gen Med. 2022;15:8557‐8565. 36536611 10.2147/IJGM.S390929 PMC9759001

[34] Li R , Shi PA , Liu TF , et al. Role of 3D psuedocontinuous arterial spin‐labeling perfusion in the diagnosis and follow‐up in patients with herpes simplex encephalitis. Am J Neuroradiol. 2019;40:1901‐1907. doi:10.3174/ajnr.A6279 31649156 PMC6975109

[35] Noguchi T , Yakushiji Y , Nishihara M , et al. Arterial spin‐labeling in central nervous system infection. Magn Reson Med Sci. 2016;15:386‐394. doi:10.2463/mrms.mp.2015-0140 27001393 PMC5608113

[36] Sathyanathan BP , Ravichandran A , Ranganathan R . ASL perfusion in atypical Japanese encephalitis. Indian J Radiol Imaging. 2020;30:536‐539. doi:10.4103/ijri.IJRI_268_20 33737791 PMC7954167

[37] Wong AMC , Yeh CH , Lin JJ , et al. Arterial spin‐labeling perfusion imaging of childhood encephalitis: correlation with seizure and clinical outcome. Neuroradiology. 2018;60:961‐970. doi:10.1007/s00234-018-2062-9 30046856

[38] Jung K , Moon HJ . A case of NMDAR encephalitis treated in the third trimester – novel arterial spin labeling findings and a review of literature. J Neuroimmunol. 2020;343:1‐6. doi:10.1016/j.jneuroim.2020.577235 32279021

[39] Li R , Jin S , Wang Y , et al. Brain perfusion alterations on 3D pseudocontinuous arterial spin‐labeling MR imaging in patients with autoimmune encephalitis: a case series and literature review. Am J Neuroradiol. 2022;43:701‐706. doi:10.3174/ajnr.A7478 35393361 PMC9089268

[40] Miao A , Liu Q , Li Z , et al. Altered cerebral blood flow in patients with anti‐NMDAR encephalitis. J Neurol. 2020;267:1760‐1773. doi:10.1007/s00415-020-09747-x 32130498

[41] Sachs JR , Zapdka ME , Popli GS , et al. Arterial spin labeling perfusion imaging demonstrates cerebral hyperperfusion in anti‐NMDAR encephalitis. Radiol Case Report. 2017;12:833‐837. doi:10.1016/j.radcr.2017.06.004 PMC5823289 29484082

[42] Sandweiss AJ , Kannan V , Desai NK , Kralik SF , Muscal E , Fisher KS . Arterial spin labeling changes parallel asymmetric perisylvian and perirolandic symptoms in 3 pediatric cases of anti‐NMDAR encephalitis. Neurol Neuroimmunol Neuroinflamm. 2023;10:e200119. doi:10.1212/NXI.0000000000200119 37094999 PMC10136681

[43] Takafumi W , Mori H , Shindo K . Serial assessment of multimodality imaging in anti‐leucine‐rich glioma‐inactivated 1 antibody encephalitis: a case report. eNeurologicalSci. 2022;29:1‐4. doi:10.1016/Jj.ensci.2022.100426 PMC9494171 36161067

[44] Yedavalli VS , Hamam O , Bahouth M , et al. Arterial spin labeling imaging characteristics of anti‐leucine‐rich glioma‐inactivated 1 encephalitis: a qualitative and quantitative analysis. Front Neur. 2022;13:1‐11. doi:10.3389/fneur.2022.850029 PMC9377014 35979060

[45] Banwell B , Bennett JL , Marignier R , et al. Diagnosis of myelin oligodendrocyte glycoprotein antibody‐associated disease: international MOGAD Panel proposed criteria. Lancet Neurol. 2023;22:268‐282. doi:10.1016/S1474-4422(22)00431-8 36706773

[46] Charpentier H , Roux CJ , Leroux P , et al. Spectrum of neuroradiological manifestations in primary hemophagocytic lymphohistiocytosis: a comparative study of EBV‐induced versus non‐EBV‐induced forms in 75 genetically confirmed pediatric cases. Eur Radiol. 2023;33:7149‐7159. doi:10.1007/s00330-023-09649-2 37171488

[47] Khoury MN , Gheuens S , Ngo L , Wang X , Alsop DC , Koralnik IJ . Hyperperfusion in progressive multifocal leukoencephalopathy is associated with disease progression and absence of immune reconstitution inflammatory syndrome. J Neurol. 2013;136:3441‐3450. doi:10.1093/brain/awt268 PMC3808691 24088807

[48] Kumar S , Nagesh CP , Thomas B , Radhakrishnan A , Menon RN , Kesavadas C . Arterial spin labeling hyperperfusion in Rasmussen's encephalitis: is it due to focal brain inflammation or a post‐ictal phenomenon? J Neuroradiol. 2017;45:6‐14. doi:10.1016/j.neurad.2017.08.002 28923528

[49] Carandini T , Sacchi L , Bovis F , et al. Distinct patterns of MRI lesions in MOG antibody disease and AQP4 NMOSD: a systematic review and meta‐analysis. Mult Scler Relat Disord. 2021;54:103118. doi:10.1016/j.msard.2021.103118 34246019

[50] Alisauskaite N , Wang‐Leandro A , Dennler M , et al. Conventional and functional magnetic resonance imaging features of late subacute cortical laminar necrosis in a dog. J Vet Intern Med. 2019;33:1759‐1765. doi:10.1111/jvim.15526 31120629 PMC6639491

[51] Hoffmann AC , Ruel Y , Gnirs K , et al. Brain perfusion magnetic resonance imaging using pseudocontinuous arterial spin labeling in 314 dogs and cats. J Vet Intern Med. 2021;35:2327‐2341. doi:10.1111/jvim.16215 34291497 PMC8478041

[52] Braund KG . Granulomatous meningoencephalomyelitis. J Am Vet Med Assoc. 1985;186:138‐141. 3882646

[53] Levine JM , Fosgate GT , Porter B , Schatzberg SJ , Greer K . Epidemiology of necrotizing meningoencephalitis in Pug dogs. J Vet Intern Med. 2008;22:961‐968. doi:10.1111/j.1939-1676.2008.0137.x 18647157 PMC7166975

[54] Cooper JJ , Schatzberg SJ , Vernau KM , et al. Necrotizing meningoencephalitis in atypical dog breeds: a case series and literature review. J Vet Intern Med. 2014;28:198‐203. 24428322 10.1111/jvim.12233 PMC4895549

[55] Nessler JM , Oevermann A , Schawacht M , et al. Concomitant necrotizing encephalitis and granulomatous meningoencephalitis in four toy breed dogs. Front Vet Sci. 2022;1(9):957285. doi:10.3389/fvets.2022.957285 PMC9477003 36118343

[56] Adamo PF , Adams WM , Steinberg H . Granulomatous menigoencephalomyelitis in dogs. Compend Contin Educ Pract Vet. 2007;29:678‐690. 18210978

[57] Barnes Heller HL , Granick MN , Pinkerton ME , Keuler NS . Case‐control study of risk factors for granulomatous meningoencephalomyelitis in dogs. J Am Vet Med Assoc. 2019;254:822‐825. 30888272 10.2460/javma.254.7.822

[58] Lawn RW , Harcourt‐Brown TR . Risk factors for early death or euthanasia within 100 days of diagnosis in dogs with meningoencephalitis of unknown origin. Vet J. 2022;287:105884. 35987308 10.1016/j.tvjl.2022.105884

[59] Smith PM , Stalin CE , Shaw D , Granger N , Jeffery ND . Comparison of two regimens for the treatment of meningoencephalomyelitis of unknown etiology. J Vet Intern Med. 2009;23:520‐526. 19645837 10.1111/j.1939-1676.2009.0299.x

[60] Ostrager A , Bentley RT , Lewis MJ , Moore GE . Survival in dogs with meningoencephalitis of unknown etiology with and without lesions detected by magnetic resonance imaging. J Vet Intern Med. 2024;38:2204‐2213. doi:10.1111/jvim.17109 38804716 PMC11256124

[61] von Praun F , Matiasek K , Grevel V , et al. Magnetic imaging and pathologic findings associated with necrotizing encephalitis in two Yorkshire terriers. Vet Radiol Ultrasound. 2006;47:260‐264. doi:10.1111/j.1740-8261.2006.00137.x 16700176

[62] Jayaraman K , Rangasami R , Chandrasekharan A . Magnetic resonance imaging findings in viral encephalitis: a pictorial essay. J Neurosci Rural Pract. 2018;9:556‐560. doi:10.4103/jnrp.jnrp_120_18 30271050 PMC6126294

[63] Andersen‐Ranberg E , Berendt M , Gredal A . Biomarkers of non‐infectious inflammatory CNS diseases in dogs – where are we now? Part I: meningoencephalitis of unknown origin. Vet J. 2021;273:1‐14. doi:10.1016/j.tvjl.2021.105678 34148601

[64] Deibler AR , Pollock JM , Kraft RA , Tan H , Burdette JH , Maldjian JA . Arterial spin‐labeling in routine clinical practice, part 2: hypoperfusion patterns. Am J Neuroradiol. 2008;29:1235‐1241. doi:10.3174/ajnr.A1033 18356467 PMC3397388

[65] Deibler AR , Pollock JM , Kraft RA , Tan H , Burdette JH , Maldjian JA . Arterial spin‐labeling in routine clinical practice, part 3: Hyperperfusion patterns. Am J Neuroradiol. 2008;29:1428‐1435. doi:10.3174/ajnr.A1034 18356466 PMC3397396

[66] Dinoto A , Cheli M , Ajcevic M , et al. ASL MRI and 18F‐FDG‐PET in autoimmune limbic encephalitis: clues from two paradigmatic cases. Neurol Sci. 2021;42:3423‐3425. doi:10.1007/s10072-021-05207-0 33763811

