# Supplementary material for: Exploring brain perfusion in dogs with meningoencephalitis of unknown origin: A promising role for arterial spin labeling imaging
Source: J Vet Intern Med. 2024 Dec 10;39(1):e17259. doi: 10.1111/jvim.17259 (PMC11629257; doi:10.1111/jvim.17259)
Supplement: Supplementary file 1 — Supporting Information S1: Implemented conventional MRI sequences. [file JVIM-39-e17259-s003.docx]

**Supporting Information S1:** Implemented conventional MRI sequences

Conventional MRI sequences implemented in all animals enrolled in the study included: a transverse 3-dimensional (3D) isotropic T1-weighted inversion recovery spoiled gradient recalled echo sequence (BRAVO), a transverse 2-dimensional (2D) T2-weighted fast spin echo (FSE) sequence or a transverse 3D isotropic T2-weighted FSE sequence (Cube T2), a dorsal 3D fluid-attenuated inversion recovery sequence using a FSE readout (Cube FLAIR), a transverse 2D 3-directional diffusion-weighted imaging (DWI) sequence with an apparent diffusion coefficient (ADC) map, a 3D T2*-based spoiled gradient echo using a multiecho acquisition named SWAN and a sagittal gadolinium-enhanced (gadolinium 0.1 mmol/L, IV - Clariscan^TM^ GE 0.5 mmol/mL, GE Healthcare SAS, France) 3D fat-saturated T1-weighted FSE sequence (Cube FS FSE T1 gado).

Parameter settings of conventional sequences are provided in the table below.

| **Sequence name** | **TR** | **TE** | **Slice thickness** | **Matrix size** | **Pixels’ size** |
| --- | --- | --- | --- | --- | --- |
| BRAVO (T1) | 7.4 ms | 3.1 ms | 1.4 mm | 200x200 | 0.7x0.7 |
| 2D FSE T2 | 5306 ms | 102 ms | 2 mm | 352x300 | 0.4x0.5 |
| Cube T2 | 1272 ms | 116 ms | 0.8 mm | 300x300 | 0.6x0.6 |
| Cube FLAIR | 7000 ms | 145 ms | 1 mm | 200x200 | 0.9x0.9 |
| Diffusion | 4285 ms | 79 ms | 3.5 mm | 100x60 | 1.0x1.0 |
| SWAN | 51 ms | 40 ms | 1.6 mm | 160x160 | 0.9x0.9 |
| Cube FS FSE T1 gado | 658 ms | 14 ms | 0.9 mm | 256x256 | 0.6x0.6 |
