# Supplementary material for: Exploring brain perfusion in dogs with meningoencephalitis of unknown origin: A promising role for arterial spin labeling imaging
Source: J Vet Intern Med. 2024 Dec 10;39(1):e17259. doi: 10.1111/jvim.17259 (PMC11629257; doi:10.1111/jvim.17259)
Supplement: Supplementary file 2 — Supporting Information S2: Arterial spin labeling sequence settings. [file JVIM-39-e17259-s002.docx]

**Supporting Information S2:** Arterial spin labeling sequence settings

Arterial spin labeling sequence settings included: 80 transverse partitions of 4 mm thickness; FOV, 240x240 mm acquisition matrix, 8 spiral arms in each 3D partition and 512 points per arm; TE, 10.6 ms; TR, 4310 ms; PLD, 1025 ms, 1525 ms or 2025 ms; NEX, 4; flip angle, 155°; acquisition time, from 5 min 19 sec to 5 min 53 sec depending on selected PLD. The caudal border of the ASL imaging slab was placed just caudal to the cerebellum.
