# Supplementary material for: Exploring brain perfusion in dogs with meningoencephalitis of unknown origin: A promising role for arterial spin labeling imaging
Source: J Vet Intern Med. 2024 Dec 10;39(1):e17259. doi: 10.1111/jvim.17259 (PMC11629257; doi:10.1111/jvim.17259)
Supplement: Supplementary file 3 — Supporting Information S3: Clinical information of 31 dogs with MUO. [file JVIM-39-e17259-s001.docx]

**Supporting Information S3:** Clinical information of 31 dogs with MUO

|  | **Total** |
| --- | --- |
|  | (n = 31) |
| **Duration of signs prior to diagnosis** |  |
| Median duration (IQR; range), days | 7 (3-15; 0.5-120) |
| Peracute (< 2 days) | 5/31 (16%) |
| Acute (2 to 7 days) | 14/31 (45%) |
| Chronic (> 7 days) | 11/30 (36%) |
| Missing data | 1/31 (3%) |
| **Clinical manifestations at initial presentation** |  |
| Deficit of cranial nerves | 16/31 (52%) |
| *Visual deficit* | *12/31 (39%)* |
| *Nasal sensitivity deficit* | *2/31 (6%)* |
| *Abnormal pupillary reflex* | *6/31 (19%)* |
| *Facial paralysis* | *1/31 (3%)* |
| Ataxia | 14/31 (45%) |
| Painful state | 9/31 (29%) |
| Reluctant to walk, climb or jump | 7/31 (23%) |
| Head tilt or head turn | 7/31 (23%) |
| Seizures | 6/31 (19%) |
| Desorientation | 5/31 (16%) |
| Circling | 5/31 (16%) |
| Tremors | 3/31 (10%) |
| Altered mentation | 3/31 (10%) |
| Abnormal behaviour | 3/31 (10%) |
| Paresis | 2/31 (6%) |
| **Time between last seizure and MRI-T0** |  |
| Median time (range), hours | 12 (7-24) |
| **CSF analysis** |  |
| Total nucleated cell count > 5/mm^3^ | 28/31 (90%) |
| Negative for *Neospora caninum* antigens | 31/31 (100%) |
| Negative for distemper antigens | 2/2 (100%) |
| **Treatments before MRI-T0** |  |
| No treatment | 25/31 (81%) |
| Corticosteroids alone | 5/31 (16%) |
| Corticosteroids and antibiotics | 1/31 (3%) |
| Dosage of corticosteroids (mg/kg/j) | .5-1 |
| Median duration of corticosteroids treatment (IQR), days | 4 (1-8.5) |

Abbreviations: CSF, Cerebrospinal fluid; IQR, Interquartile range; MRI, Magnetic resonance imaging; MUO, Meningoencephalitis of unknown origin
